# Multiple tachykinins and their receptors characterized in the gastropod mollusk Pacific abalone: Expression, signaling cascades, and potential role in regulating lipid metabolism

**DOI:** 10.3389/fendo.2022.994863

**Published:** 2022-09-12

**Authors:** Seungheon Lee, Mi Ae Kim, Jong-Moon Park, Keunwan Park, Young Chang Sohn

**Affiliations:** ^1^ Department of Marine Bioscience, Gangneung-Wonju National University, Gangneung, South Korea; ^2^ East Coast Life Sciences Institute, Gangneung-Wonju National University, Gangneung, South Korea; ^3^ College of Pharmacy, Gachon University, Incheon, South Korea; ^4^ Natural Product Informatics Research Center, KIST Gangneung Institute of Natural Products, Gangneung, South Korea

**Keywords:** neuropeptide, tachykinin, GPCR, evolution, lipid synthesis, invertebrate

## Abstract

Tachykinin (TK) families, including the first neuropeptide substance P, have been intensively explored in bilaterians. Knowledge of signaling of TK receptors (TKRs) has enabled the comprehension of diverse physiological processes. However, TK signaling systems are largely unknown in Lophotrochozoa. This study identified two TK precursors and two TKR isoforms in the Pacific abalone *Haliotis discus hannai* (Hdh), and characterized Hdh-TK signaling. Hdh-TK peptides harbored protostomian TK-specific FXGXRamide or unique YXGXRamide motifs at the C-termini. A phylogenetic analysis showed that lophotrochozoan TKRs, including Hdh-TKRs, form a monophyletic group distinct from arthropod TKRs and natalisin receptor groups. Although reporter assays demonstrated that all examined Hdh-TK peptides activate intracellular cAMP accumulation and Ca^2+^ mobilization in Hdh-TKR-expressing mammalian cells, Hdh-TK peptides with N-terminal aromatic residues and C-terminal FXGXRamide motifs were more active than shorter or less aromatic Hdh-TK peptides with a C-terminal YXGXRamide. In addition, we showed that ligand-stimulated Hdh-TKRs mediate ERK1/2 phosphorylation in HEK293 cells and that ERK1/2 phosphorylation is inhibited by PKA and PKC inhibitors. In three-dimensional *in silico* Hdh-TKR binding modeling, higher docking scores of Hdh-TK peptides were consistent with the lower EC50 values in the reporter assays. The transcripts for *Hdh-TK* precursors and *Hdh-TKR* were highly expressed in the neural ganglia, with lower expression levels in peripheral tissues. When abalone were starved for 3 weeks, *Hdh-TK1* transcript levels, but not *Hdh-TK2*, were increased in the cerebral ganglia (CG), intestine, and hepatopancreas, contrasting with the decreased lipid content and transcript levels of sterol regulatory element-binding protein (*SREBP*). At 24 h post-injection *in vivo*, the lower dose of Hdh-TK1 mixture increased *SREBP* transcript levels in the CG and hepatopancreas and accumulative food consumption of abalone. Higher doses of Hdh-TK1 and Hdh-TK2 mixtures decreased the *SREBP* levels in the CG. When *Hdh*-*TK2*-specific siRNA was injected into abalone, intestinal *SREBP* levels were significantly increased, whereas administration of both *Hdh*-*TK1* and *Hdh-TK2* siRNA led to decreased *SREBP* expression in the CG. Collectively, our results demonstrate the first TK signaling system in gastropod mollusks and suggest a possible role for TK peptides in regulating lipid metabolism in the neural and peripheral tissues of abalone.

## Introduction

Tachykinins (TKs) are one of the most extensively studied neuropeptide families. They were found mainly in the neurons and intestines of bilaterians (1). The major mammalian TKs are the first neuropeptide substance P (SP), neurokinin A (NKA) and neurokinin B (NKB), together with NH_2_-terminally extended forms of NKA, including neuropeptide K (NPK) and neuropeptide γ (NPγ) (2). TK family members are encoded by three *TAC* genes: *TAC1* encoding SP, NKA, NPK, and NPγ; *TAC3* (*TAC2* in rodents) encoding NKB; and *TAC4* encoding hemokinin-1 and endokinins A–D (1, 2). Most vertebrate and ascidian (chordate-type) TK peptides have a consensus sequence of FXGLMamide at the C-terminus, whereas protostomes TK peptides have a C-terminal FX_1_GX_2_Ramide sequence (3–7). A sister group of the TK peptides, termed natalisins (NTLs) having a C-terminal FX_1_X_2_X_3_Ramide sequence, was subsequently found in the Panarthropoda lineage (comprising arthropods, onychophores, and tardigrades) (1, 8).

The tissue distribution of TK peptides and their precursors was studied to reveal the presumptive function and the synthesis process of the TKs in diverse animal phyla. As with other neuropeptides, TK peptides are mainly produced by neurons in the central nervous system (CNS) and neural ganglia and by enteroendocrine cells associated with the intestine tissues (1), such as the brain mushroom bodies of the honey bee (*Apis mellifera*) (7), the midgut endocrine cells of the crab (*Cancer magister*) (5) and the fly (*Drosophila melanogaster*) (9, 10), and the visceral ganglia of the oyster (*Crassostrea gigas*) (11). They are widely distributed in various tissues and exert pleiotropic effects throughout diverse species in the animal kingdom. For example, TKs play roles in stimulation of intestinal muscle contraction and tubular fluid secretion (9, 12), lipid metabolism in enterocytes (10), nociception of sensory neurons (13, 14), olfactory sensory signaling (15, 16), and social behavior (17, 18), and learning and memory circuits (19). TKs are also involved in the regulation of reproductive activity (20, 21), pheromone detection in a gustatory neural circuit (22), and controlled release of hormones, such as insulin (23). Interestingly, there are protostomian TK peptides with a chordate-type FXGLMamide or FXGMRamide motif at the C-terminus. These are produced by toxin (salivary or venom) glands of the yellow fever mosquito (*Aedes aegypti*), the parasitoid Jewel wasp (*Nasonia vitripennis*), and the common octopus (*Octopus vulgaris*) (24–26). These findings suggest that the invertebrate venom TK peptides are exogenously delivered to “prey” animals, acting on their receptors in the circuits of the CNS, peripheral nervous systems, and smooth muscles in the ileum and hindgut to lead paralysis, vasodilation, and contraction (1, 24, 27). These protostomian chordate-type TK peptides with a C-terminal FXGLMamide or FXGMRamide motif are, for simplicity, hereafter referred to as “exocrine” TKs.

One or more TK receptors (TKRs) mediate the actions of TK peptides. In vertebrates, three TKRs, NK1R–NK3R (or TAC1R–TAC3R), were characterized as seven transmembrane G protein-coupled receptors of the rhodopsin-like class A family and are mainly found in the CNS and peripheral nervous systems (1). SP and other TK peptides, such as NKA and NKB, have differential affinity and selectivity for the three TKRs, as follows: SP > NKA > NKB for NK1R, NKA > NKB > SP for NK2R, and NKB > NKA > SP for NK3R (2, 28). Signaling through the vertebrate TKRs is diverse and complex: for example, the ligand-bound NK1R activates 1) phospholipase C (PLC), inducing the mobilization of intracellular Ca^2+^ and the activation of protein kinase C (PKC); 2) adenylyl cyclase (AC), increasing intracellular cyclic adenosine monophosphate (cAMP) accumulation and activation of protein kinase A (PKA); 3) phospholipase A2, generating arachidonic acids and prostaglandins (2).

The protostomes TKRs have been characterized in a wide range of phyla, such as arthropods, annelids, mollusks, and even cnidarians, which are considered the most basal metazoans. However, many are orphan receptors for which no ligand has been identified (1, 29). The functional roles and signaling of TKRs, including arthropod-specific NTL receptors (NTLRs), are extensively studied in Ecdysozoa (8, 18, 30–32). Functional TKRs have been reported in three species of Lophotrochozoa: annelid *Urechis unitinctus* (33), cephalopod mollusk *O. vulgaris* (34), and bivalve mollusk *C. gigas* (11). However, to the best of our knowledge, TKR signaling has not yet been investigated in gastropod mollusks. Since Gastropoda is the largest and most diverse class of the phylum Mollusca (35), elucidating TK signaling and functions in gastropod mollusks may provide in-depth information on the molecular conservation throughout evolution and diversified regulatory pathways of TKR system in bilaterians.

To analyze the physiological roles of TK in gastropod mollusks, we first characterized a specific TK signaling system in Pacific abalone (*Haliotis discus hannai*; Gastropoda; Mollusca). This study demonstrates that the TK peptides activate the abalone TKRs at nanomolar concentrations, triggering both PKC/Ca^2+^ and PKA/cAMP signal-transduction pathways. In addition, we determined the potential involvement of the TK signaling network in the control of lipid production in the Pacific abalone through a sterol regulatory element-binding protein (SREBP), as cellular lipid metabolism and homeostasis are globally controlled by the transcription factor SREBP (36).

## Materials and methods

### Nucleotide/amino acid sequences and phylogenetic analyses

Two sequences for TK precursors were previously determined as entire nucleotide sequences in the Pacific abalone *H. discus hannai* (Hdh) transcriptome databases (37, 38). Signal peptides, cleavage sites, and predicted mature peptide sequences of Hdh-TK1 and Hdh-TK2 precursors (NCBI GenBank accession numbers MZ197811, MZ197812) were analyzed by SignalP-5.0 (http://www.cbs.dtu.dk/services-/SignalP) and Neuropred (http://stagbeetle.animal.uiuc.edu/cgi-bin/neuropred.py). Sequence alignments for representative bilaterian TK mature peptides were produced using Clustal Omega Multiple Sequence Alignment with default parameters (https://www.ebi.ac.uk/Tools/msa/clustalo). The open-source software BOXSHADE was used to highlight conserved amino acids.

To identify homologous sequences for TKR in the Hdh transcriptome databases (37, 38), we employed Basic Local Alignment Search Tool (BLAST; https://blast.ncbi.nlm.nih.gov/Blast.cgi) with the sequences of molluscan TKRs (11, 34). Prediction of the transmembrane helices in the abalone TKRs, Hdh-TKRL (long isoform) and Hdh-TKRS (short isoform), was performed using the TMHMM program (http://www.cbs.dtu.dk/services/TMHMM). The *N*-linked glycosylation and intracellular phosphorylation sites were predicted by the NetNGlyc and NetPhos servers (http://www.cbs.dtu.dk/services).

In order to generate the phylogenetic tree, amino acid sequences of TKRs were retrieved from references (1, 11) and the NCBI non-redundant sequence repository. Phylogenetic tree analysis was performed as previously described (39). Briefly, the amino acid sequences of TKRs and related receptors were aligned using MUSCLE and trimmed using the online tool NGPhylogeny (https://ngphylogeny.fr/). The trimming contained 293 residues for the receptors used to generate the maximum likelihood tree using W-IQ server (40). The substitution models, LG+F+I+G4, the ultrafast bootstrap approximation approach, and SH-aLRT 1000 replicates were used. Phylogenetic trees were visualized using FigTree software.

### cDNA cloning and plasmid construction

In May 2017, female abalone [8.5 ± 0.5 cm shell length; 80.9 ± 8.0 g body weight (BW)] were purchased from a local dealer (Gangneung, Gangwon-do, Korea). Total RNAs were extracted from the cerebral ganglion (CG) and pleuro-pedal ganglion (PPG) using the RNeasy Mini kit (Invitrogen, Waltham, MA, USA), and first-strand cDNAs were synthesized using PrimeScript RT reagent kit with gDNA Eraser (Takara, Osaka, Japan) per the manufacturer’s instructions. Polymerase chain reaction (PCR) was performed using the synthesized CG and PPG cDNAs as templates, PrimeSTAR HS DNA polymerase (Takara), and oligo primer sets (**Table 1**). The PCR cycling conditions were as follows: 1 min at 98°C; 35 cycles of 10 s at 98°C, 15 s at 54°C, 1.5 min at 72°C; and 5 min at 72°C. The PCR-amplicons were digested by EcoRI and XbaI, and then cloned into the restriction enzyme sites of the HA-tagged pcDNA3 expression vector (Invitrogen). The plasmid constructs were analyzed to verify the correct sequence by Sanger sequencing.

**Table 1:** Oligo primer sequences used in the polymerase chain reaction.

Target	Direction	Sequence (5′–3′)	Application
*Hdh-TKRL*	Sense	CGCGGAATTCATGGCAGAGTTAACTACTACTATC	cDNA cloning
*Hdh-TKRL*	Antisense	CGCGTCTAGATTAAACAGAATCATCCCGCGT	
*Hdh-TKRS*	Sense	CGCGGAATTCATGGCAGAGTTAACTACTACTATC	
*Hdh-TKRS*	Antisense	CGCGTCTAGATTAAGCAAGGGTTACAGAACC	
*Hdh-TK1* precursor	Sense	CGCGGGCTACAGGAGGAAC	RT-qPCR
*Hdh-TK1* precursor	Antisense	ACGTAACAAAGGCGGTGGCA	
*Hdh-TK2* precursor	Sense	CTCCCAACAGAGGGCGCATC	
*Hdh-TK2* precursor	Antisense	CTGGCCCTCGACAACCGTTA	
*Hdh-TKR*	Sense	GTGCCATTTCTGTTAGTGTTCTG
*Hdh-TKR*	Antisense	CACGTCTTACTGGCAGGATT	
*Hdh-SREBP*	Sense	CCTTGCTCGCTACTTCCTAAG	
*Hdh-SREBP*	Antisense	TGAGCCAACCACTGAACATG	
*Hdh-RPL5*	Sense	TCACCAACAAGGACATCATTTGTC	
*Hdh-RPL5*	Antisense	CAGGAGGAGTCCAGTGCAGTATG	
*Hdh-TK1* precursor	Sense	GCCAAGGTGTGATATTTGTACTG	siRNA validation
*Hdh-TK1* precursor	Antisense	CCGCTTCTCTGCTACTGTTG
*Hdh-TK2* precursor	Sense	GATGCTCCTTGAGAACCGAG	
*Hdh-TK2* precursor	Antisense	CCTTAACCTCTGGAGCCATG	

Underlined nucleotides indicate restriction enzyme recognition sites.

### Peptide extraction and LC-MS/MS analysis

The hemolymph and the neural ganglia from the CG and PPG were prepared from four adult abalone and used for the peptide extraction process. Peptide extraction and mass spectrometry were performed as previously described (37), with slight modifications. The acquired MS/MS raw files were searched using Proteome discoverer™ 2.5 software (PD), applying the abalone TBI unigene of the ganglia protein sequence database (37). The workflow included a precursor ions quantifier step, a peptide validator step, and SEQUEST HT process for detection as a database search algorithm. The following search parameters were set: precursor abundance based on intensity; 20 ppm of tolerances of precursor ion mass; 0.02 Da fragment ion mass; no-enzyme (unspecific); peptide length of at least six amino acids. The dynamic modifications of the peptide sequence were as follows: oxidation of methionine (+15.995 Da), dioxidation of methionine (+31.990 Da), carbamylation of cysteine (+43.006 Da), acetylation of N-terminus (+42.011 Da), cyclization of N-terminal glutamic acid to pyro-glutamic acid (–18.011 Da), cyclization of N-terminal glutamine to pyro-glutamic acid (–17.027 Da), and amidation of C-terminus (–0.984 Da).

### Peptide synthesis

Predicted mature peptides derived from Hdh-TK1 and Hdh-TK2 precursors were synthesized by Anygen Co., Ltd. (Gwangju, Korea) with a purity of >96% as analyzed by high-performance liquid chromatography (**Table 2**).

**Table 2:** Synthesized Hdh-TK peptide sequences.

Peptide	Sequence
Hdh-TK1-1	FGYVGSR-amide
Hdh-TK1-2	TELGFGYVGSR-amide
Hdh-TK1-3	pQPHFGFHGVR-amide
Hdh-TK2-1	GRHFGFVGSR-amide
Hdh-TK2-2	KPHFGFHGSR-amide

### Luciferase reporter assay

Human embryonic kidney 293 (HEK293) cells were maintained in Dulbecco’s modified Eagle medium (DMEM; Gibco, Loughborough, UK) containing 10% fetal bovine serum (FBS; Hyclone, GE Healthcare, Chicago, IL, USA) and 1% penicillin/streptomycin (P/S; Invitrogen) at 37°C in 5% CO_2_. Sixteen hours before transfection, HEK293 cells were seeded into 24-well plates (5 × 10^4^ cells/well). Transfection and luciferase activities were analyzed as previously described (39). Briefly, HEK293 cells were transfected with 100 ng of pcDNA3-HA expression plasmid containing Hdh-TKR (pcDNA3-HA-Hdh-TKR) or vehicle pcDNA3-HA plasmid using 0.9 μL of a polyethyleneimine transfection reagent (Sigma-Aldrich, St. Louis, MO, USA), along with 100 ng of a luciferase reporter plasmid containing cAMP response element (CRE-Luc) or serum response element (SRE-Luc), and 100 ng of a pRSV-β-galactosidase expression plasmid as an internal control. At 3 h after transfection, the culture medium was replaced by new DMEM with 1% P/S and 10% FBS, and the HEK293 cells were cultured for a further 30 h and then maintained in DMEM without FBS for 16 h. Finally, the HEK293 cells were treated with synthesized peptides, forskolin (Sigma-Aldrich), 12-O-tetradecanoylphorbol-13-acetate (TPA; Sigma-Aldrich), or an equal volume of peptide-free medium (as vehicle control) for 6 h. Luciferase activity was analyzed using a microplate-luminometer (Berthold Tech., Bad Wildbad, Germany) and normalized to the β-galactosidase activity, detected by absorbance at 405 nm using a microplate reader (Tecan, Mannedorf, Switzerland).

### Ca^2+^ mobilization assay

Ca^2+^ mobilization assays were performed as described previously (39). Briefly, Chinese hamster ovary K1 (CHO-K1) cells were maintained in DMEM-F12 Nutrient Mixture Ham supplemented with 10% FBS (Hyclone) and 1% P/S (Invitrogen) at 37°C in 5% CO_2_. Cells were grown in a monolayer to 60% confluency in 100 mm dishes (1.5 × 10^6^ cells) and transiently transfected with 2 μg of pcDNA3-HA-Hdh-TKR plasmid with 2 μg of a Ca^2+^-reporter aequorin plasmid and 2 μg of Gαq plasmid using 18 μL of FuGENE6 transfection reagent (Promega). The cells were washed in assay buffer including phenol red-free DMEM/F-12 with 0.1% bovine serum albumin (BSA) and 1% P/S and collected by brief centrifugation. Probenecid (1.25 mM final concentration, Invitrogen) and coelenterazine (5 μM, Gold Biotech., St. Louis, MO, USA) were added to resuspended CHO-K1 cells in 5 mL of assay buffer and the cells were gently agitated for 2.5 h using a magnetic stirrer at room temperature and shielded from light. Just before the Ca^2+^ mobilization assay, peptide solutions were prepared in the assay buffer, and 50 μL aliquots were dispensed into 3 or 4 wells of a 96-well microplate for each peptide. While stirring gently, 50 μL of the cell suspension was injected into a luminescence microplate reader (Berthold Tech.), and luminescence was recorded for 20 s and for a further 10 s after injection of the assay buffer, including 0.2% Triton X-100 to measure the total Ca^2+^ response. Dose-response curves and half maximal effective concentrations (EC50) values were obtained using Sigma Plot v.13 (Systat Software, San Jose, CA, USA).

### Western blotting

HEK293 cells were seeded into 6-well plates (0.3 × 10^6^ cells/well) and transfected with 3 μg of pcDNA3-HA-Hdh-TKR plasmids or vehicle pcDNA3-HA plasmid as described for the luciferase reporter assays. At 24 h after transfection, the cells were incubated with serum-free DMEM for 6 h at 37°C and then exposed to a PKA inhibitor (H89, 10^-5^ M; Sigma-Aldrich) or PKC inhibitor (Gö6983, 10^-5^ M; Sigma-Aldrich) for 60 min. After the medium was replace with fresh serum-free DMEM, the cells were exposed to Hdh-TK-2-2 peptide (5×10^-9^ or 10^-7^ M) or TPA (10^-7^ M) for a further 5 min. The methods employed for cell lysate preparation, polyacrylamide gel electrophoresis, protein transfer to nitrocellulose membranes, and membrane washing were from a previous report (39). The membranes were incubated with a monoclonal mouse p44/42 MAPK, rabbit phospho-p44/42 MAPK (ERK1/2) (Thr202/Tyr204) (Cell Signaling, Danvers, MA, USA), or mouse GAPDH (Santa Cruz Biotechnology, Santa Cruz, CA, USA) primary antibody at 4°C for 16 h, followed by incubation with goat anti-mouse IgG-HRP or goat anti-rabbit IgG-HRP secondary antibody (Santa Cruz Biotechnology, Santa Cruz, CA, USA) at room temperature for 2 h. After incubation with a chemiluminescence western blotting substrate reagent (Thermo Fisher Scientific, Waltham, MA, USA) at room temperature for 5 min, the reactive bands were visualized using a C-Digit 3600 Bolt Scanner (LI-COR, Lincoln, NE, USA).

### Homology modeling and peptide docking for Hdh-TKRL

Homology modeling for Hdh-TKRL structure was performed by SWISS-MODEL (41). The active human NK1R (PDB ID 7RMG) (42) was selected as a modeling template as it had the highest sequence identity (45.5%). The peptide docking was then performed by HPEPDOCK Server (43) to predict the binding mode of peptides in the Hdh-TKRL model structure. The Hdh-TK peptide binding site in Hdh-TKRL was inferred from the SP binding site found in the template structure (42). The Hdh-TK peptide sequences without chemical modification were docked for modeling peptide binding complex structures. The conformation with the best docking score was selected to analyze peptide–receptor interactions.

### Experimental Abalone and Designs

#### Tissue distribution of Hdh-TK precursor and Hdh-TKR transcripts

In September 2020, adult mature female and male *H. discus hannai* (n=10 for each sex; 9.0 ± 0.5 cm shell length; 90.2 ± 9.0 g BW) were purchased from a local dealer (Gangneung, Gangwon-do, Korea). The CG, PPG, hepatopancreas, gonads, intestine, gills, adduct muscle, and mantle were dissected and immediately frozen in liquid nitrogen before storage at −80°C. The tissues were analyzed by real-time quantitative PCR (RT-qPCR).

#### Different feeding conditions: Fed and food-deprived abalone

In February and March 2021, adult female abalone (n = 70; 6.9 ± 0.5 cm shell length; 30.2 ± 5.0 g BW) were purchased from a local dealer (Gangneung, Gangwon-do, Korea) and maintained in a flow-through seawater aquarium (17–20°C; 400 L) for 1 week with an *ad libitum* diet of kelp (*Saccharina japonica*). To reduce bias based on individual variation, the abalone that did not merely consume the kelp were excluded from this experiment. Subsequently, the abalone were divided into fed and food-deprived (starved) groups and then maintained for further 3 weeks. The CG, PPG, hepatopancreas, ovary, and intestine were dissected and immediately frozen in liquid nitrogen and kept at −80°C before RT-qPCR analysis.

#### Hdh-TK peptide injection

In February 2022, adult abalone (n = 150; 6.0 ± 0.5 cm shell length; 25.0 ± 3.0 g BW) were purchased from a local dealer (Gangneung, Gangwon-do, Korea), maintained in a flow-through seawater aquarium (17–20°C; 400 L) for 1 week with an *ad libitum* diet of kelp (*S. japonica*). Abalone that did not merely consume the kelp were excluded from this experiment. Subsequently, the abalone were randomly divided into six groups (n = 12 per group) and allowed a diet of kelp *ad libitum* for a further week. Abalone were weighed before each assay, and 200 μL of mollusk saline (13 g HEPES, 25.66 g NaCl, 0.82 g KCl, 1.69 g CaCl_2_, 10.17 g MgCl_2_, 2.56 g Na_2_SO_4_, 1.0 L dH_2_O; pH 7.2) including Hdh-TK1 peptide mixture, Hdh-TK2 peptide mixture, or APGWamide, was injected into the adduct muscle sinus using a 1 mL syringe with a 26-gauge needle. Low doses of Hdh-TK1 and Hdh-TK2 peptides and high doses of peptides, including APGWamide, were set at 0.25 and 2.5 μg/peptide/g BW, respectively. Control abalone were injected with an equal volume of mollusk saline. Injected abalone were placed in individuals (30 × 12 × 7.5 cm) and supplied with seawater-immersed kelp equivalent to 7% of BW. Food intake was assessed at 24 h after injection, as described previously (39). The CG, hepatopancreas, and intestine were dissected and immediately frozen in liquid nitrogen and kept at −80°C before RT-qPCR analysis.

#### Injection of small interfering RNA against *Hdh-TK* precursors

siRNA duplexes against *Hdh-TK1* and *Hdh-TK2* precursors were designed and produced by Bioneer Inc. (Daejeon, Korea; **Table 3**), and AccuTarget™ green fluorescent protein (GFP) siRNA (Bioneer Inc.) was used as control siRNA. The sequences of siRNAs and oligo primers for transcript validation are presented in **Table 1**. Pacific abalone (25.79 ± 0.6 g BW; n = 40) were maintained in a flow-through seawater aquarium (17–18°C; 400 L) for 2 days with an *ad libitum* diet of kelp. Abalone were administered as 50 μg siRNA in 100 μl of phosphate-buffered saline (pH 7.2; Thermo Fisher Scientific) by injection into the adduct muscle sinus (n = 10 per group). At 44 h post-injection, CG and intestine tissues were dissected, immediately frozen in liquid nitrogen, and kept at −80°C before RT-qPCR analysis.

**Table 3:** siRNAs used in the RNA interference assay.

Target	Sense (5´–3´)	Antisense (5´–3´)
*Hdh-TK1* precursor	GACUCGAAGGAACGUCUGU	ACAGACGUUCCUUCGAGUC
*Hdh-TK2* precursor	CUGUAGACACGACACAGUU	AACUGUGUCGUGUCUACAG

### RT-qPCR analysis

Based on the Minimum Information for Publication of Quantitative Real-Time PCR Experiments (MIQE) guidelines (44), transcript levels of target genes were validated using RT-qPCR, as previously described (38, 45). Briefly, total RNA was extracted using the RNeasy Mini Kit (Invitrogen), and 1 μg of RNA was reverse-transcribed to first-strand cDNA using the PrimeScript RT reagent kit (Takara). Ribosomal protein L-5 (*Hdh-RPL-5*) mRNA was selected and used as an internal reference as previously described (38, 45). SYBR-based RT-qPCR was performed on the Quant Studio 7 Flex Real-Time PCR System (Applied Biosystems, CA, USA) using the following reaction conditions: 95°C for 30 s, followed by 40 cycles of 95°C for 5 s and 60°C for 34 s. **Table 1** lists the primer sets used in this study. The relative mRNA expression levels were calculated from the formula: 2^–(Ct target gene–Ct reference gene)^. The specificities of the primer pairs were confirmed by melting curve analyses at the end of each RT-qPCR run.

### Triglyceride measurements

TG levels were analyzed by the TG quantitation kit (ab65336, abcam; Waltham, MA, USA) from the abalone intestine according to the manufacturer’s protocol. Briefly, lipids were extracted by homogenizing 100 mg of intestine tissues in 1 mL of 5% NP-40 solution using a pestle and mortar and then slowly heated to 95°C in the water bath for 5 min. The samples were cooled, heated to solubilize all TGs into solution, and centrifuged at 15,200 *g* force for 2 min at room temperature. The supernatant was diluted 10 times with double-distilled water, and lipase and TG reaction mixture were added as the kit supplier recommended. The mixed samples were incubated at room temperature for 1 h while protected from light, and the absorbance was analyzed on a microplate reader (EL800, Bioteck, Vermont (VT), USA) at 562 nm.

### Statistical analysis

Statistical significance was determined by one-way analysis of variance (ANOVA) followed by Tukey’s *post-hoc* test or unpaired Student’s *t*-test when the data were normally distributed. In some cases, data were log-transformed before analysis to meet the parametric assumptions of normality and equal variance using SPSS v.25.0 software (SPSS Inc., Chicago, IL, USA). Differences were considered statistically significant for *p* values of < 0.05.

## Results

### Sequence analysis of Hdh-TK precursors and mature peptides

**Figure 1:**
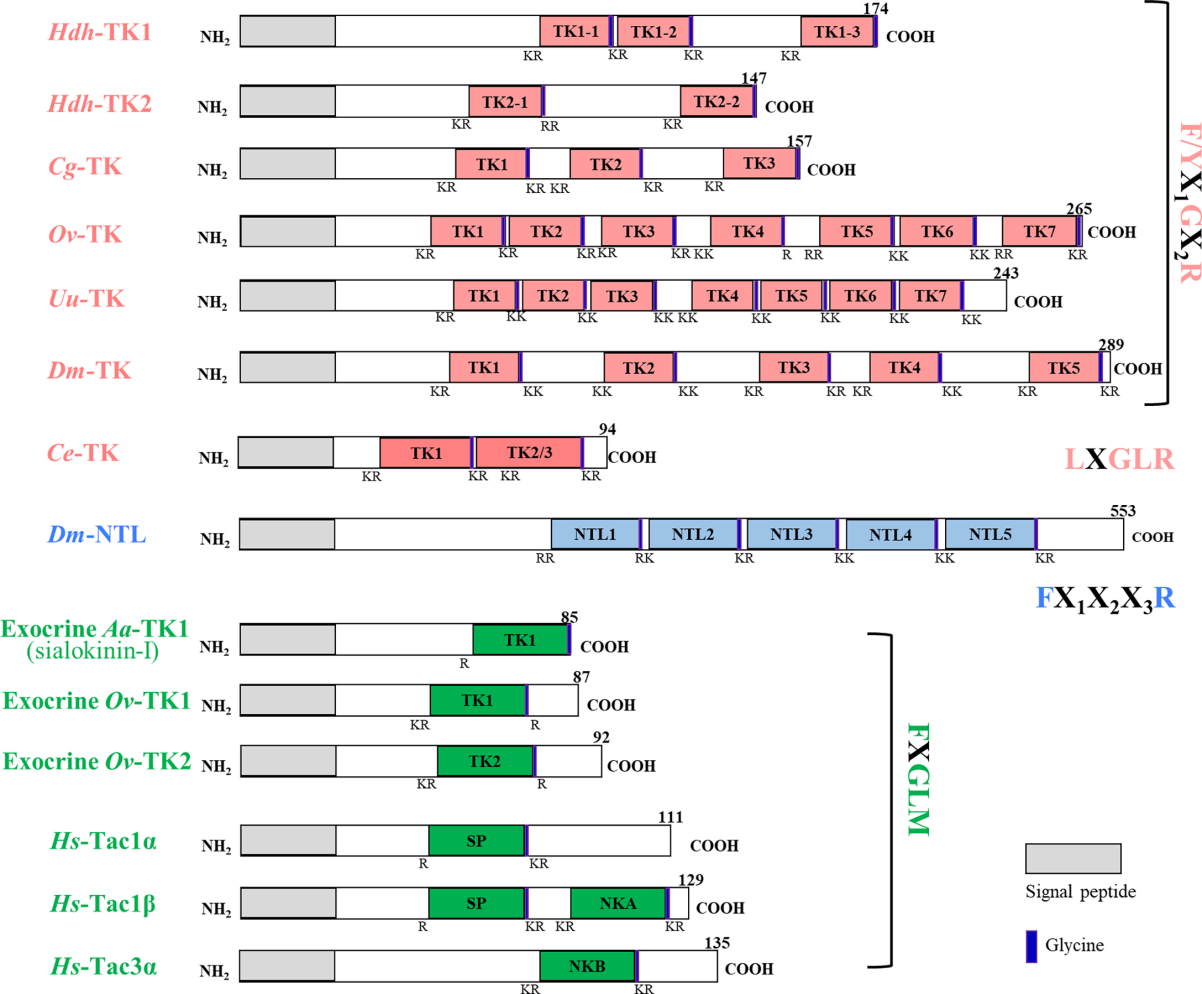
Schematic representation of protosome TK precursors and their human homologs. The signal peptide and glycine are denoted by the gray box and purple bar, respectively. The nucleotide and deduced amino acid sequences of the *H. discus hannai* TK precursors, *Hdh*-TK1 and *Hdh*-TK2, are available in **Supplementary Figures 1A, B**, respectively. **Supplementary Table 1** provides information on the sequences.

The nucleotide length for the *Hdh-TK1* precursor was 1877 bp, including a 5′-untranslated region (UTR), coding sequence (CDS) for 174 amino acids, and a 3′-UTR. The *Hdh-TK2* precursor was 2078 bp long, including a CDS for 147 amino acids (**Figure 1** **;** **Supplementary Figures 1A, B** **)**. The two Hdh-TK precursors contain N-terminal signal peptides with 15 amino acids, multiple repeats of mature Hdh-TK peptides consisting of 7–11 amino acids with a G residue, responsible for biological amidation, followed by dibasic cleavage signals (combination of R and K) (**Supplementary Figures 1A, B** **)**. The protostomian TK and NTL precursors encode multiple peptide paracopies (isoforms) on the same precursor, whereas the exocrine TK precursors encode a single copy of the TK peptide (**Figure 1**). Hdh-TK1 peptides harbor one C-terminal consensus FX_1_GX_2_Ramide and two analogous YX_1_GX_2_Ramide motifs, whereas Hdh-TK2 peptides contain the FX_1_GX_2_Ramide motif only. The Y-type Hdh-TK1 peptides are unique throughout the characterized TK peptides in deuterostomes and protostomes (**Supplementary Figure 2**). The nano-LC-MS/MS spectrometry analysis detected Hdh-TK1-2, -TK1-3, and -TK2-1 peptides with the predicted modifications from the hemolymph and/or the neural ganglia of Pacific abalone (**Supplementary Figure 3**). Other predicted Hdh-TK1-1 and TK2-2 peptides were not detected in this analysis.

### Sequence analysis of Hdh-TKRs

**Figure 2:**
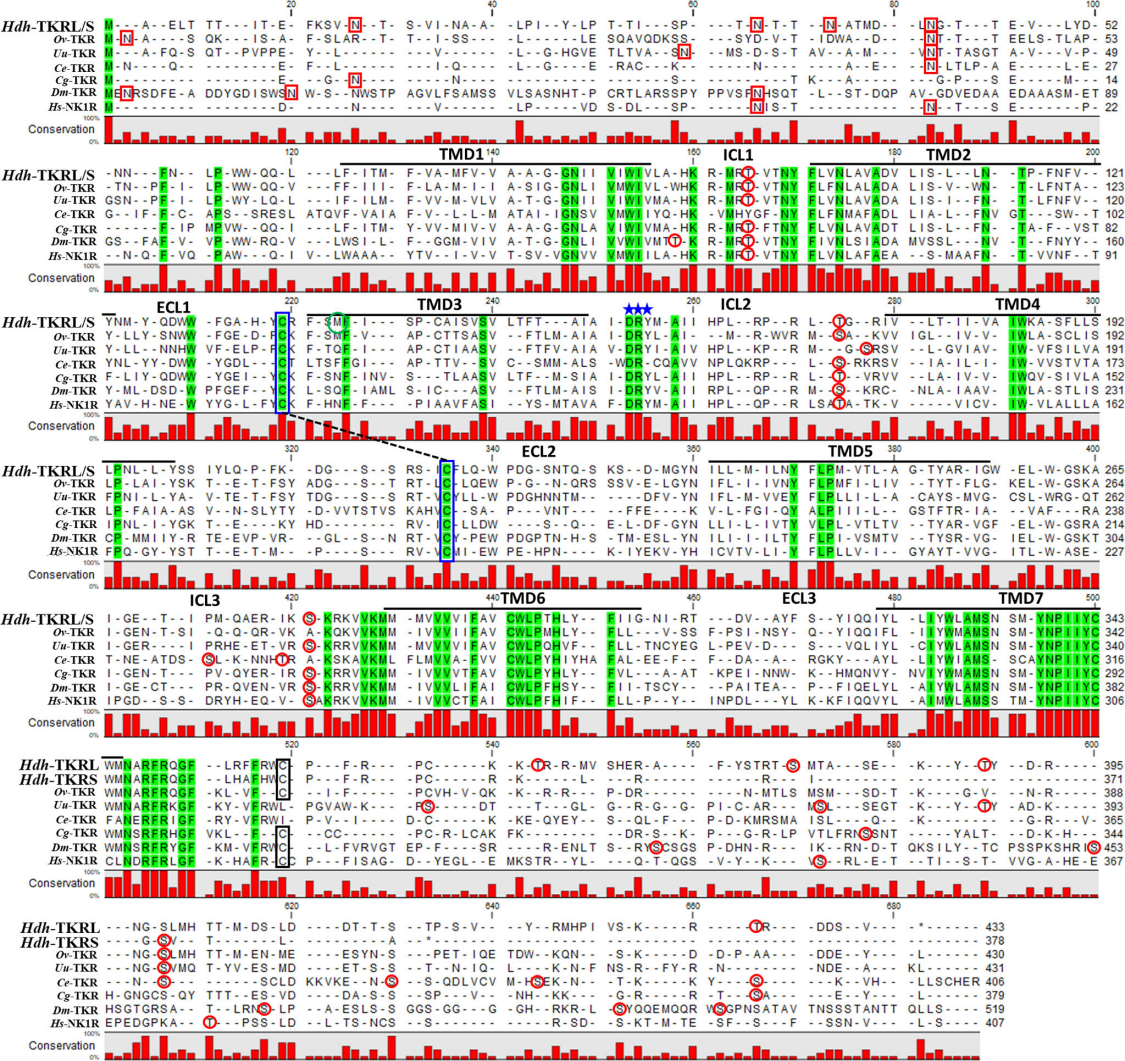
Amino acid sequence alignment of Hdh-TKRs with representative functional TKRs. The predicted seven transmembrane domains (TMD1–7) between intracellular loops (ICL1–3) and extracellular loops (ECL1–3) are indicated above the alignment. Amino acid residues that are common to these receptors are highlighted in green. Potential *N*-linked glycosylation sites, the characteristic DRY/F sequence of rhodopsin-like GPCR, and consensus PKC and PKA phosphorylation sites are denoted with red boxes, blue stars, and red circles, respectively. Conserved cysteine residues forming a disulfide bond between ECL1 and ECL2 TMD3 are indicated by blue boxes with a dotted line. Black-boxed cysteine residues indicate the consensus palmitoylation sites. The amino acid sequences of *Hdh*-TKRL (NCBI accession number MW810094) and *Hdh*-TKRS (MW929758) were placed in a multiple alignment with those of *Octopus vulgaris*-TKR (*Ov*-TKR: AB096700), *Urechis unicinctus*-TKR (*Uu*-TKR: BAB87199.1), *Caenorhabditis elegans*-TKR (*Ce*-TKR: NP_499064.2), *Crassostrea gigas*-TKR (*Cg*-TKR: XP_022673516.1), *Drosophila melanogaster*-TKR (*Dm*-TKR: X62711.1), and *Homo sapiens*-NK1R (*Hs*-NK1R: AAA59936.1).

The predicted abalone TKR sequences that displayed homology with protostome TKRs, Hdh-TKRL and Hdh-TKRS, had characteristics of the GPCR family: one N-terminal extracellular domain (ECD), seven transmembrane domains (TMDs), three extracellular loops (ECLs) and intracellular loops (ICLs), and one C-terminal intracellular domain (ICD) (**Figure 2**). Similar to other rhodopsin-like GPCRs, the DRY motif in ICL2, two C-residues forming a disulfide bond between the ECL1 and ECL2, and a C-residue in the ICD for palmitoylation were highly conserved across bilaterian TKRs (**Figure 2**). Both Hdh-TKRL and Hdh-TKRS had putative consensus glycosylation sequences (N-X-S/T) in the ECDs and multiple PKA/PKC-phosphorylation sequences (R/KXS/T; R/KX_1_X_2_S/T) in the ICLs and C-terminal ICDs.

**Figure 3:**
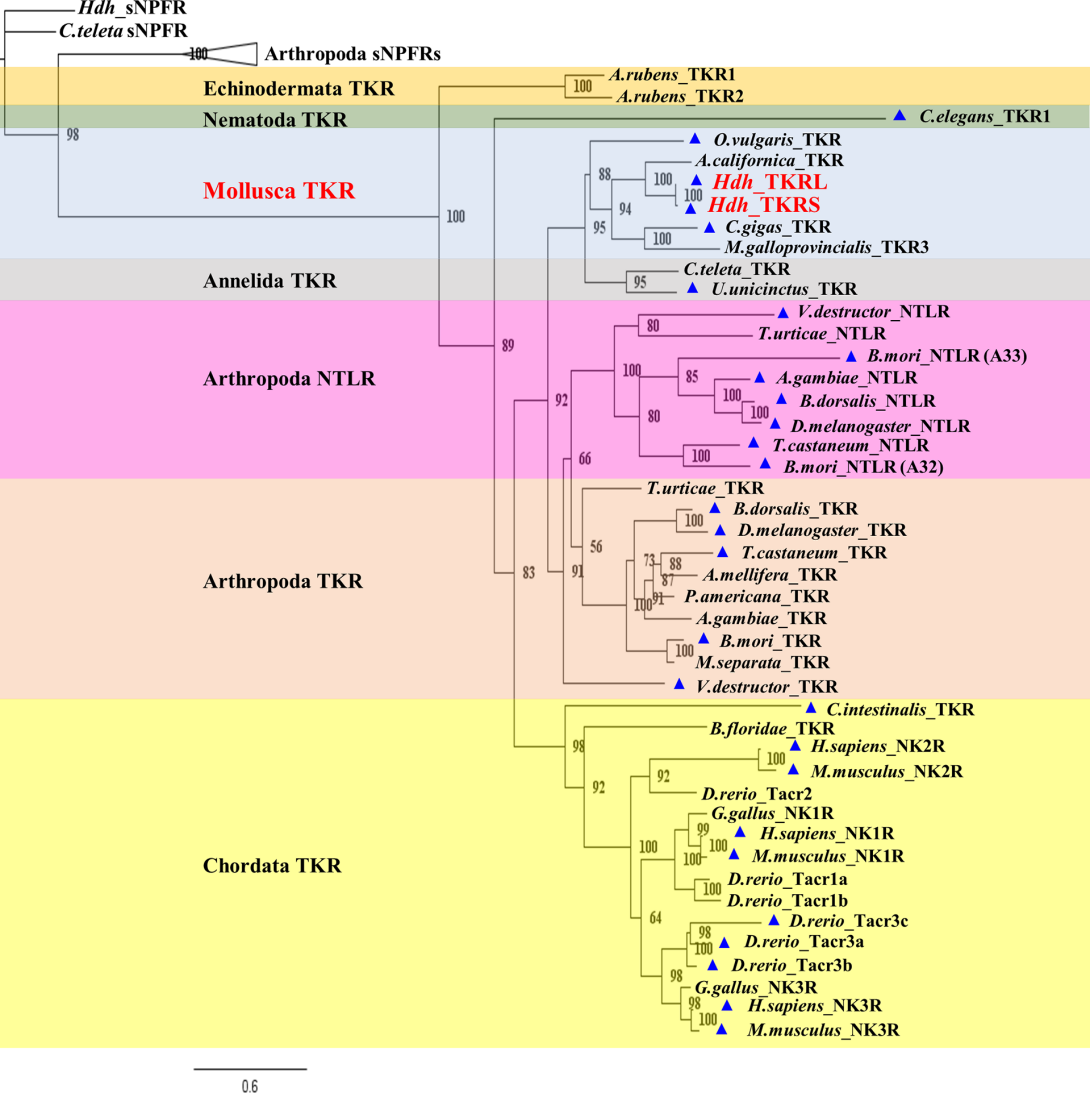
Phylogenetic tree analysis of Hdh-TKRs with bilaterian TKRs and arthropod-specific NTLRs. The numbers at the branches show the bootstrap probability after 1000 iterations in constructing the tree. The lower scale bar represents 0.6 amino acid replacements per site. The blue triangle indicates receptors that have been functionally characterized. **Supplementary Table 2** provides information for sequences used to generate the phylogeny.

To investigate relationships of the Hdh-TKRs with other bilaterian TKRs and related receptors, we performed a phylogenetic analysis using the NCBI database with bilaterian TKRs and sNPF receptors as an outgroup. This analysis revealed that Hdh-TKRL and Hdh-TKRS form a clade with the gastropod *A. californica* TKR and that gastropod TKRs share a recent common ancestor with bivalve TKRs **(** **Figure 3**). In addition, mollusk and annelid TKRs are a sister group to arthropod TKR/NTLR groups, which form a distinct clade of chordate TKRs.

### Signaling *via* Hdh-TKRs

**Figure 4:**
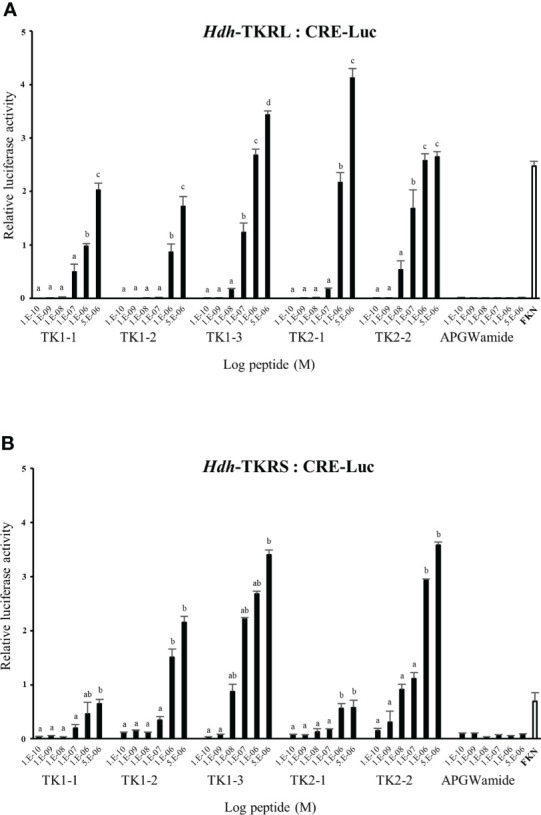
CRE luciferase reporter activities in **(A)** Hdh-TKRL- and **(B)** Hdh-TKRS-expressing HEK293 cells treated with Hdh-TK peptides. Hdh-APGWamide peptide was used as a negative control. The relative activities were determined in response to 10^−5^ M of forskolin (FKN) and various concentrations of TK peptides and APGWamide. The relative reporter activities are presented as the mean ± SEM (n = 3). Lower case letters indicate statistically significant differences (p < 0.05).

**Figure 5:**
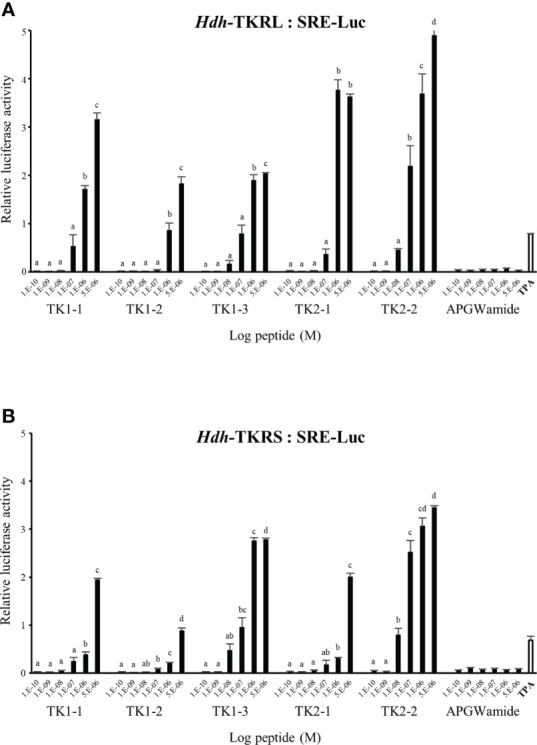
SRE luciferase reporter activities in **(A)** Hdh-TKRL- and **(B)** Hdh-TKRS-expressing HEK293 cells treated with Hdh-TK peptides. Hdh-APGWamide was used as a negative control. The relative activities were determined in response to 10^−7^ M of 12-O-tetradecanoylphorbol-13-acetate (TPA) and various concentrations of TK peptides and APGWamide. The relative reporter activities are presented as the mean ± SEM (n = 3). Lower case letters indicate statistically significant differences (p < 0.05).

**Figure 6:**
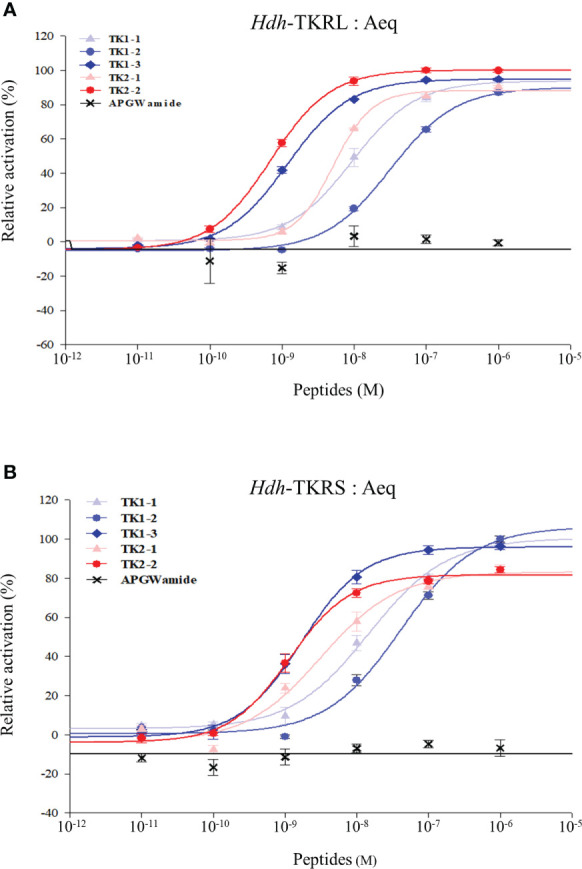
Dose-response curves for intracellular Ca^2+^ mobilization in Hdh-TKR- and aequorin (Aeq)-expressing CHO-K1 cells. Luminescence was plotted relative to the maximal response achieved when TK peptides (10^−6^ M) were applied to the **(A)** Hdh-TKRL- or **(B)** Hdh-TKRS-transfected CHO-K1 cells. APGWamide was used as the negative control. Data represent the mean ± SEM (n = 3 or 4).

To evaluate the signaling pathways involved in abalone TKs, luciferase reporter systems were applied to determine Ca^2+^ mobilization and cAMP accumulation in Hdh-TK peptide-stimulated Hdh-TKRL- or Hdh-TKRS-expressing mammalian cells. In general, all examined Hdh-TK peptides derived from Hdh-TK1 and Hdh-TK2 precursors increased the SRE-Luc and CRE-Luc activities in the Hdh-TKRs-transfected HEK293 cells in a dose-dependent manner, whereas abalone APGWamide did not activate the reporter activities in the same context (**Figures 4**, **5**). Analysis of the inhibitory pathway using the CRE-Luc system revealed that even higher concentrations of Hdh-TK peptides (5 μM) could not inhibit the forskolin-stimulated CRE-Luc activities in Hdh-TKR-expressing HEK293 cells (**Supplementary Figure 4**). However, there was no CRE-Luc and SRE-Luc reporter activity in the Hdh-TK peptide-stimulated HEK293 cells transfected with pcDNA3 vehicle plasmid **(** **Supplementary Figures 5, 6**). Hdh-TKR-transfected CHO-K1 cells stimulated a luminescent response of the Ca^2+^ reporter aequorin to Hdh-TK peptides in a dose-dependent manner, regardless of the presence of Gα15 (**Figure 6** **;** **Supplementary Figure 7**). Specifically, Hdh-TK1-3, -TK2-1, and -TK2-2, with the conserved C-terminal FX_1_GX_2_Ramide motif, showed stronger activation of Hdh-TKRs with EC50 of 0.7–5.2 nM compared with Hdh-TK1-1 and -TK1-2 containing YX_1_GX_2_Ramide (EC50 of 9.4–46.0 nM; **Table 4**). However, there was no luminescence response of Ca^2+^ reporter aequorin in the Hdh-TK peptide-stimulated CHO-K1 cells transfected with pcDNA3 vehicle plasmid (**Supplementary Figure 8**).

**Table 4:** Half-maximal effective concentrations (EC50) of Hdh-TK peptides on intracellular Ca^2+^ mobilization.

Peptide	EC50 (nM)	Receptor
Hdh-TK1-1	9.4	Hdh-TKRL
Hdh-TK1-2	32.0	
Hdh-TK1-3	1.1
Hdh-TK2-1	5.2	
Hdh-TK2-2	0.7	
Hdh-TK1-1	14.7	Hdh-TKRS
Hdh-TK1-2	46.0	
Hdh-TK1-3	1.7
Hdh-TK2-1	4.0	
Hdh-TK2-2	1.2	

**Figure 7:**
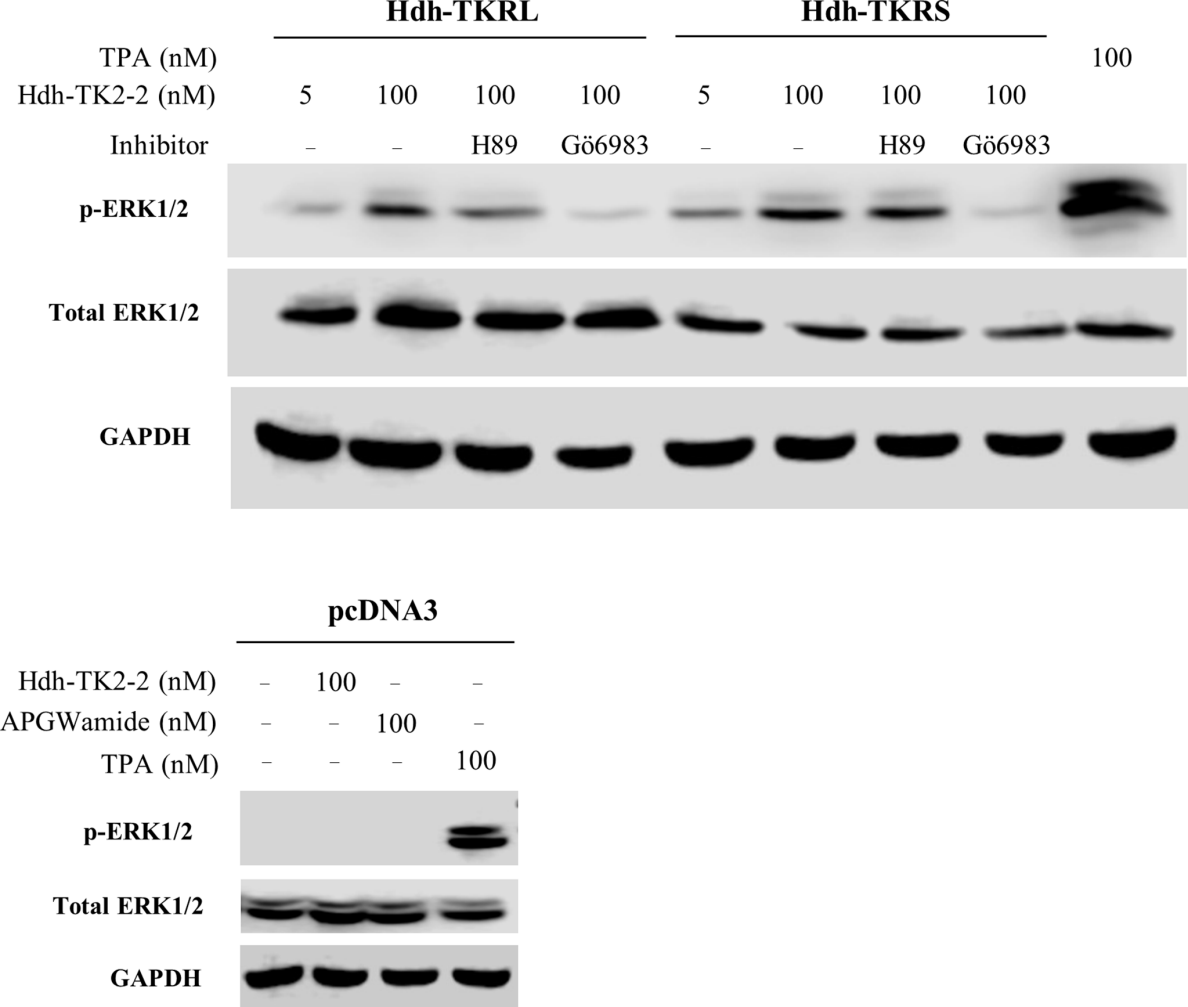
Effects of PKA inhibitor H89 and PKC inhibitor Gö6983 on Hdh-TKRL- or Hdh-TKRS-mediated activation of ERK1/2 in HEK293 cells. The cells were pre-incubated with or without inhibitors (10^−5^ M) for 1 h and then stimulated with Hdh-TK2-2 or 12-O-tetradecanoylphorbol-13-acetate (TPA) at the indicated concentrations for 5 min. Phospho-ERK1/2 (p-ERK1/2), total ERK1/2, and GAPDH expression were examined by western blotting of whole-cell lysates. As a control, HEK293 cells transfected with the maternal plasmid, pcDNA3, were also stimulated with Hdh-TK2-2, APGWamide, or TPA at the indicated concentrations, and ERK and GAPDH expression were examined.

Hdh-TK2-2 treatment increased ERK phosphorylation in the Hdh-TKRL- and TKRS-transfected HEK293 cells in a concentration-dependent manner (**Figure 7**). The Hdh-TKRL- and TKRS-mediated phosphorylation of ERK1/2 was almost abolished by the PKC inhibitor Gö6983. Treatment with the PKA inhibitor H89 decreased the phosphorylation of ERK1/2 in Hdh-TKRL-transfected HEK293 cells and to a lesser extent in Hdh-TKRS-transfected cells.

### *In silico* modeling of abalone TK-TKR complex

**Figure 8:**
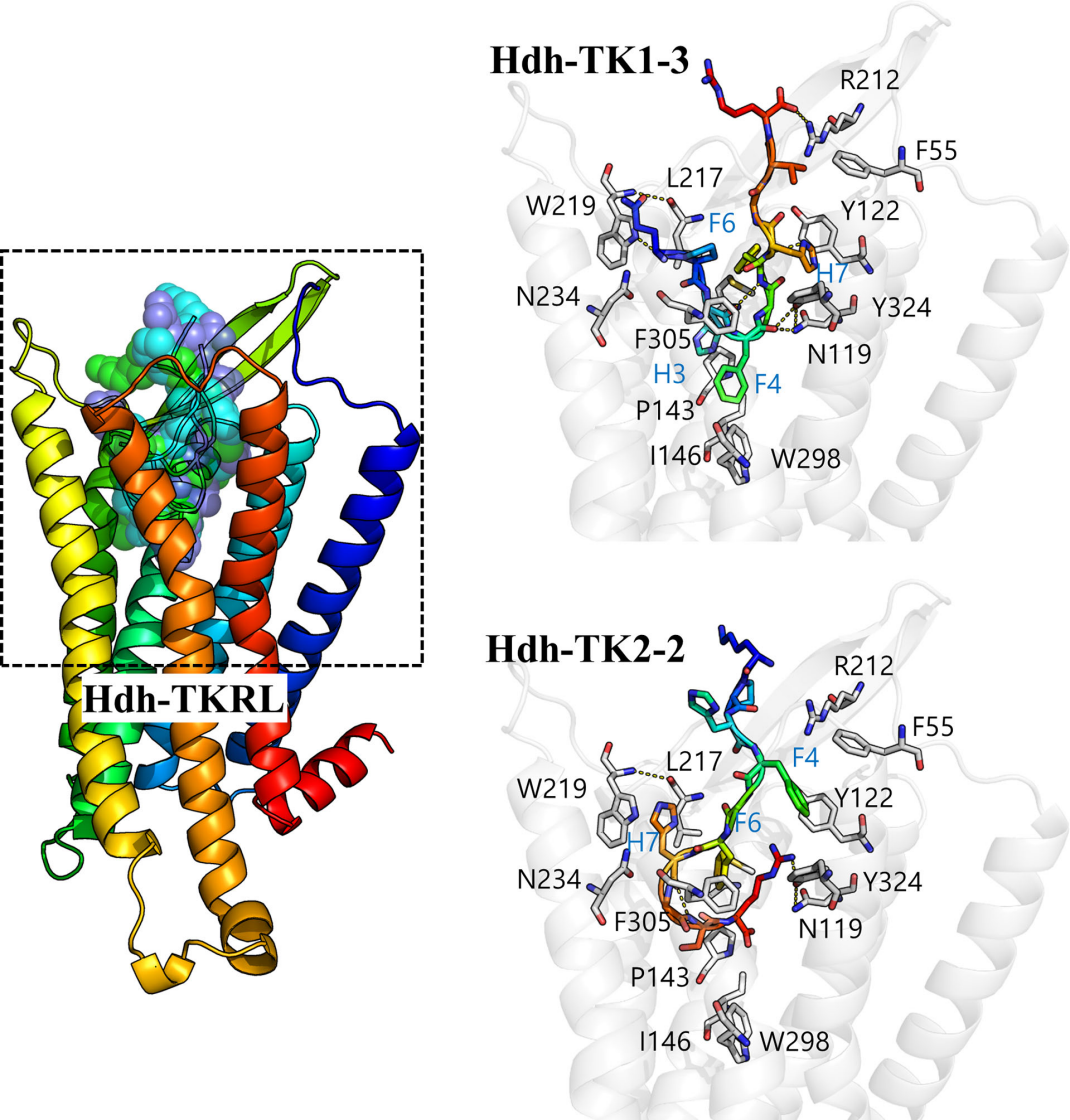
*In silico* docking model of the Hdh-TK–TKR complex. A color gradient represents the bound Hdh-TK1-3/TK2-2 peptide and receptor structures from blue (N-terminus) to red (C-terminus). The peptide binding interface (black dashed box) is enlarged to show the details of the residue interactions. The receptor backbone and binding site residues are shown in white. Yellow dotted lines represent the hydrogen bonds.

We performed docking simulation for the five Hdh-TK peptides to predict potential binding modes of TK peptides and estimate the critical interactions in the binding pocket of Hdh-TKRL. The resulting docking scores were –229.57, –221.10, –271.14, –283.54, and –271.08 Rosetta energy units for Hdh-TK1-1, -TK1-2, -TK1-3, -TK2-1, and -TK2-2, respectively. While the Hdh-TK1-3, -TK2-1, and -TK2-2 peptides represented comparable docking scores, the less effective peptide Hdh-TK1-1 and -TK1-2 had markedly poorer scores. The docking models of Hdh-TK1-3 and -TK2-2 showed that hydrophobic interactions mediated by aromatic amino acids in the peptides were responsible for the variation in peptide docking scores. For example, in the Hdh-TK1-3 binding model, His3 and Phe4 in Hdh-TK1-3 are deeply inserted inside the binding pocket, forming extensive hydrophobic contacts to Pro143, Ile146, and Trp298 in Hdh-TKRL (**Figure 8**). Phe6 and His7 in Hdh-TK1-3 also represent another hydrophobic patch interacting with the aromatic amino acids in Hdh-TKRL, such as Tyr122, Leu217, and Tyr324. Hdh-TK2-2 peptide displayed similar hydrophobic interactions, namely that Phe4, Phe6, and His7 in Hdh-TK2-2 mainly interact with Phe55, Leu217, Trp219, and Phe305 in Hdh-TKRL. In contrast, the hydrophobic residues in the Hdh-TK1-2, such as Phe5 and Tyr7, form unfavorable contacts with the polar residues Asn119 and Asn234 of Hdh-TKRL, which might explain the comparatively higher EC50 value (**Table 4** **;** **Supplementary Figure 9**).

### Tissue distribution of *Hdh-TK* precursors and *Hdh-TKR* transcripts

**Figure 9:**
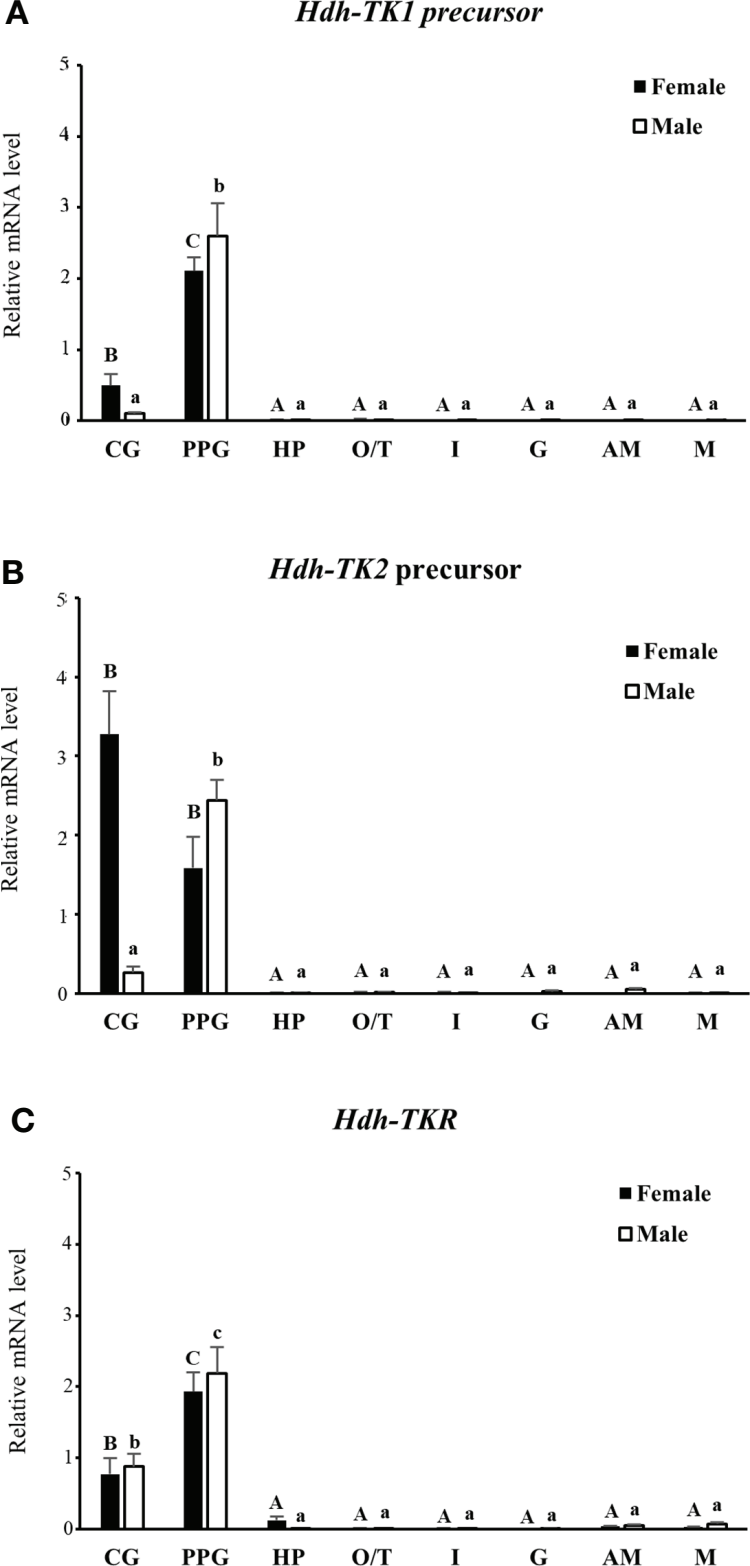
Tissue distribution of *Hdh-TK* precursors and *Hdh-TKR* transcripts in adult abalone. **(A)** Transcript levels of *Hdh-TK1* and **(B)** *Hdh-TK2* precursors, and **(C)** *Hdh-TKR* in female and male abalone were measured by real-time quantitative PCR. The ribosomal protein L-5 (*Hdh-RPL5*) was used as the internal control. All data represent the mean ± SEM (n = 6). Different upper and lower case letters on the bars indicate statistically significant differences (p < 0.05). CG, cerebral ganglion; PPG, pleuro-pedal ganglion; HP, hepatopancreas; O, ovary; T, testis; I, intestine; G, gills; AM, adduct muscle; M, mantle.

The expression patterns of the *Hdh-TK1 and Hdh-TK2* precursors and *Hdh-TKR* transcripts in adult female and male Pacific abalone tissues were analyzed by RT-qPCR. In general, the *Hdh-TK* precursors and *Hdh-TKR* mRNAs were dominantly expressed in the neural ganglia, CG and PPG, compared with those of other examined tissues in both male and female abalone (**Figure 9**). More specifically, the *Hdh-TK* precursors and *Hdh-TKR* transcripts showed significantly (*p* < 0.05) higher levels in the PPG than those in the CG, except for the *Hdh-TK2* precursor transcript in neural ganglia in females.

### Effect of food-deprivation on transcripts of *Hdh-TK* precursors, *Hdh-TKR*, and *SREBP*

**Figure 10:**
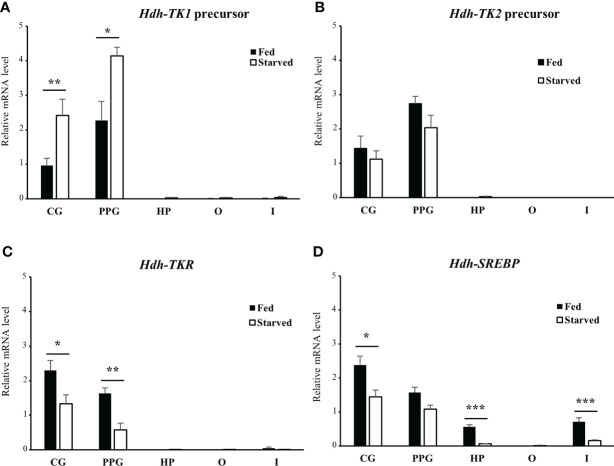
Relative transcript levels of **(A)** *Hdh-TK1* and **(B)** *Hdh-TK2* precursors, **(C)** Hdh-TKR, and **(D)** *Hdh-SREBP* in various tissues from fed or starved abalone for 3 weeks. Relative transcript levels were measured by real-time quantitative PCR. The ribosomal protein L-5 (*Hdh-RPL5*) was used as the internal control. All data represent the mean ± SEM (n = 6). Statistical significance was tested by Student’s t-test. *p < 0.05, **p < 0.01, ***p < 0.001. CG, cerebral ganglion; PPG, pleuro-pedal ganglion; HP, hepatopancreas; O, ovary; I, intestine.

To explore the possible role of TK signaling systems in lipid homeostasis in abalone, the *Hdh-TK* precursors and *Hdh-TKR* transcript profiles were analyzed for fed abalone and abalone starved for 3 weeks. In addition, *SREBP* transcript levels were determined, as SREBPs globally regulate the expression of genes involved in lipid synthesis (36). The *Hdh-TK1* precursor transcript significantly increased in the CG and PPG of starved abalone compared with those of fed abalone (p<0.05; **Figure 10A**), although there was no significant difference in the *Hdh-TK2* precursor transcript levels between fed and starved abalone (**Figure 10B**). In addition, the *Hdh-TK1* precursor transcript level was at least three times higher in the intestine and hepatopancreas of starved abalone than in fed abalone, although the differences were not significant. *Hdh-TKR* transcript levels were significantly lower in the CG and PPG of the starved abalone (p<0.05; **Figure 10C**). *Hdh-SREBP* transcript levels in the CG, hepatopancreas, and intestine significantly decreased in starved abalone (p<0.05; **Figure 10D**). As expected, the intestinal TG content was significantly lower in the starved abalone (p<0.05; **Supplementary Figure 10**).

### Effect of Hdh-TK peptides and siRNA on *SREBP* transcript

**Figure 11:**
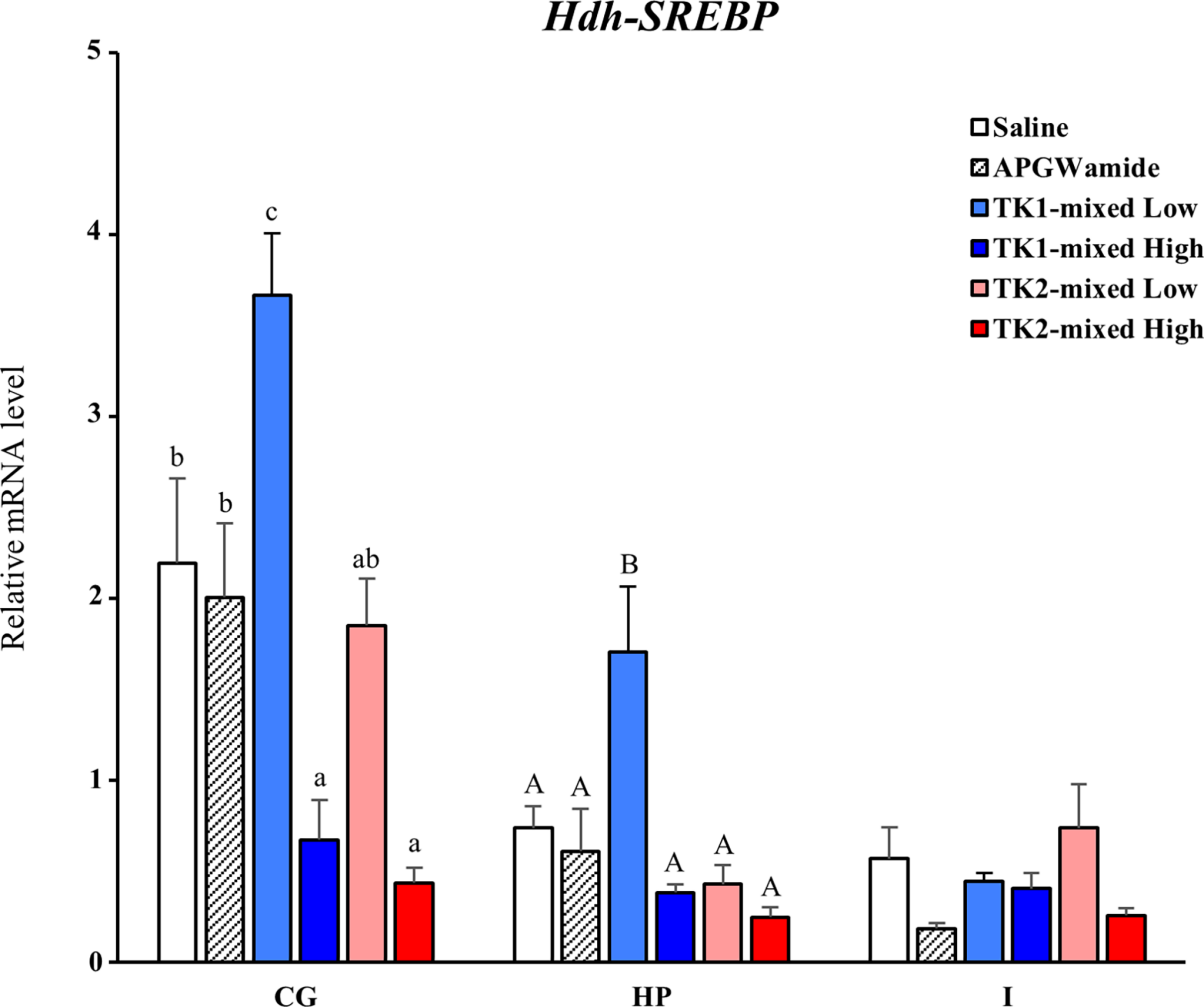
Effect of Hdh-TK peptides on relative transcript levels of *Hdh-SREBP* in abalone. At 24 h after injection of Hdh-TK1 and TK-2 peptide mixtures (low dose, 0.25 μg/g BW; high dose, 2.5 μg/g BW) along with an equal volume of saline and APGWamide (2.5 μg/g BW), the tissues of abalone were sampled. The relative *Hdh-SREBP* transcript levels were measured by real-time quantitative PCR. The ribosomal protein L-5 (*Hdh-RPL5*) was used as the internal control. All data represent the mean ± SEM (n = 8). Different upper and lower case letters on the bars indicate statistically significant differences (p < 0.05). CG, cerebral ganglion; HP, hepatopancreas; I, intestine.

**Figure 12:**
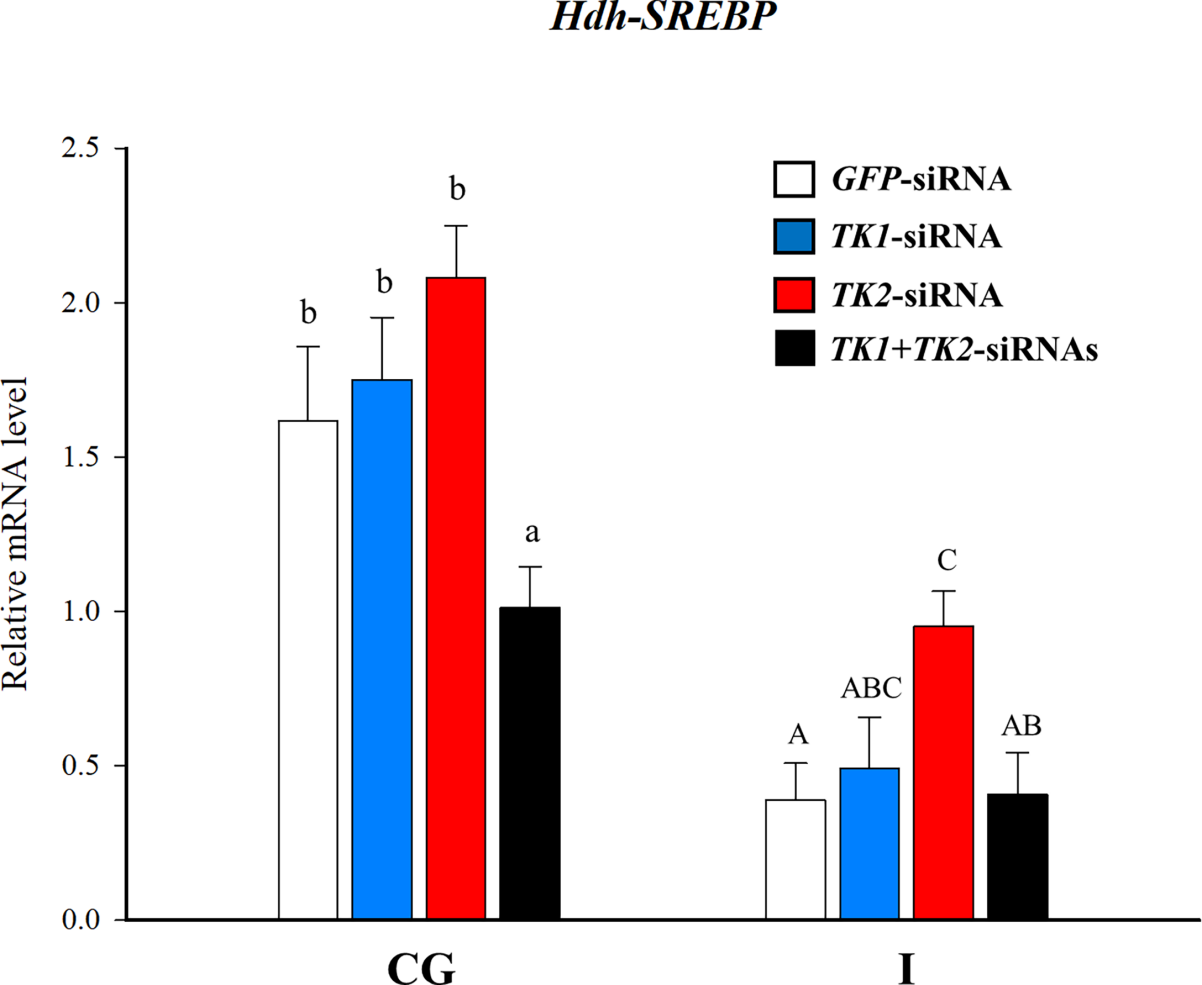
Relative expression levels of *Hdh-SREBP* in the cerebral ganglion and intestine after administration of *Hdh-TK1* and/or *Hdh-TK2* specific siRNAs (50 μg/ind.). As a control, an equal amount of *GFP*-specific siRNA was administrated. At 44 h post-injection, tissues were sampled and relative *Hdh-SREBP* transcript levels were measured by real-time quantitative PCR. All data are presented as the mean ± SEM (n = 7 or 8). Different upper and lower case letters on the bars indicate statistically significant differences (p < 0.05). CG, cerebral ganglion; I, intestine.

To reveal the direct involvement of TK peptides in lipid homeostasis in Pacific abalone, we examined a short-term effect of Hdh-TK peptides on *SREBP* transcript levels in the CG, hepatopancreas, and intestine. At 24 h after peptide injection, the lower dose of Hdh-TK1 mixture significantly (p<0.05) increased the *Hdh-SREBP* transcript levels in the CG and hepatopancreas compared with those in the saline- and APGWamide-injected abalone (**Figure 11**). In contrast, the higher dose of Hdh-TK1 or Hdh-TK2 mixture significantly (p<0.05) decreased the *Hdh-SREBP* transcript levels only in the CG. Hdh-TK peptides did not significantly change the *Hdh-SREBP* transcript levels in the intestine (**Figure 11**). Injection of abalone with the lower dose of Hdh-TK1 mixture resulted in a significant increase in food consumption compared with saline injection (p<0.05; **Supplementary Figure 11**). In addition, the knockdown effects of *Hdh-TK* precursors on *Hdh-SREBP* transcript levels were examined using *Hdh-TK1*- and *Hdh-TK2*-specific siRNA duplexes. At 44 h after siRNA injection, *Hdh-SREBP* transcript levels in the CG were significantly decreased following combined administration of *Hdh-TK1* and *Hdh-TK2* siRNA (p<0.05), whereas intestinal *Hdh-SREBP* levels were increased following administration of *Hdh-TK2* siRNA compared to *GFP* siRNA (**Figure 12**). The knockdown of the *Hdh-TK1* and *Hdh-TK2* precursor transcripts was reduced by *Hdh-TK1* siRNA and combined siRNAs for *Hdh-TK* precursors in the CG, and by *Hdh-TK1* and *Hdh-TK2* siRNAs in the intestine, significantly (p<0.05; **Supplementary Figure 12**).

## Discussion

TK signaling has various effects on physiological processes in metazoans. To the best of our knowledge, the molluscan TK receptors have been functionally characterized from only two species, *O. vulgaris* (34) and *C. gigas* (11). Although TK and TK-like neuropeptides in mollusks were first identified more than 20 years ago, the biological roles of TKs and their signaling systems were not fully elucidated in this phylum and the Lophotrochozoa if extended. The present study reports the first characterization of a TK signaling system in the Gastropoda Pacific abalone and suggests that it is functionally related to nutritional conditions. Our results provide fundamental information for further studies on the comprehensive classification, functional prediction, and modeling of molluscan neuropeptide interaction with GPCRs.

Protostomian TK peptides produced from the brain and neural ganglia display a highly conserved C-terminal FX_1_GX_2_Ramide sequence, along with distinctly classified TK-related peptides, i.e., exocrine TKs and arthropod-specific NTLs (1, 8, 26). Here, we report two distinct TK precursors with two or three paracopies harboring the consensus FX_1_GX_2_Ramide and unique YX_1_GX_2_Ramide sequences in Pacific abalone. As far as we know, the Y-type TK peptides are the first to be discovered in the TK peptide family in Bilateria and Cnidaria (1, 29). Using an analogous series of alanine-substituted *C. gigas* TK peptides, Favrel and colleagues demonstrated that only the replacement of the first F or the last R residues in the consensus FX_1_GX_2_Ramide sequence was critical for activation of the bivalve *C. gigas* TK receptor (11). Considering that the first F and Y residues are closely related and are aromatic amino acids, the two residues are possibly common, and consistent with the high degree of conservation during the evolution of protostome TK precursors. Indeed, among several arthropod NTLs with similar C-terminal FX_1_X_2_X_3_Ramide sequences, *Bombyx* NTLs with a Y-type residue instead of F residue had comparable ligand activities with their receptors (8). Nevertheless, the F-type Hdh-TK peptides had higher ligand activation in both Hdh-TKRL and Hdh-TKRS compared to Y-type Hdh-TK peptides, possibly explaining the strong selective pressure during the evolution process of TK precursor genes. Recently, in the nematode *Caenorhabditis elegans*, three TK-like peptides harboring a C-terminal LR/KGLRamide sequence showed potent agonist activities against the TKR (46). In addition, TKs, including SP, NKA, and NKB, from diverse phyla such as arthropods, mollusks, and mammals caused weak induction of the *C. elegans* TKR responses. Thus, the non-conserved N-terminal residues of various lengths might be less important than the conserved C-terminal residues. The final residue R in the protostomian TKs and NTLs is strictly conserved and important for their receptor activation. In contrast, the C-terminal M residue instead of R in exocrine TKs is unique among the protostomian TK-related peptide family and somewhat resembles deuterostomian TK peptides (8, 34, 47). The exocrine TK peptides are most likely produced from the salivary or toxin glands to act on receptors of host or prey animals, deviate structurally from endogenous TK peptides, and are encoded as a single copy by the gland-specific TK genes (1, 26). These results, including tissue distribution of TK precursor transcripts, strongly suggest that Hdh-TK1 and -TK2 peptides are genuine members of the protostomian TK family.

This study reveals the existence of TK signaling system in the gastropod mollusk. The molluscan TK receptors have been previously identified and functionally characterized in a cephalopod (*O. vulgaris*) and a bivalve (*C. gigas*) (11, 34). With a few exceptions, most protostomes have a single TK receptor with multiple TK peptides (1). The arthropod TKRs can be activated by TKs from heterospecies and, surprisingly, by the NTLs (8), suggesting that the TK peptide diversification arose first and subsequent duplication of TKR. Two abalone transcripts, *Hdh-TKRL* and *Hdh-TKRS*, had identical sequences from the start codons to the TMD7 coding regions connected to the ICD amphipathic helix. This may account for alternative splicing isoforms from a single *Hdh-TKR* gene or initial gene duplication event at the chromosome level, as we observed multiple splicing isoforms and tandem duplication of neuropeptides with their receptors in Pacific abalone (38, 39). Hdh-TKRL and Hdh-TKRS most likely belong to the superfamily of rhodopsin-like GPCRs, which are well characterized, containing a DRY sequence in the ICL2, a disulfide bridge between the two C-residues connecting the ECL1/TMD3 and ECL2, and a palmitoylated C-residue in the ICD (48, 49). In addition, we assume that the N-terminal ECD of Hdh-TKRs containing *N*-glycosylation sites and a conserved NQF-like NNF sequence play critical roles in peptide binding and intracellular signaling, as demonstrated in the ECD of human NK1R (50, 51). Hdh-TKRL has multiple PKA/PKC-phosphorylation sequences in the C-terminal ICD and ICLs, similar to other functional TKRs, which implies that the conserved residues are responsible for downstream signal transduction. Hdh-TKRS has a relatively shorter ICD, but this short ICD includes a putative PKC-phosphorylation sequence (RIGS), explaining underphosphorylation of ERK1/2 by a PKC inhibitor despite stimulation with the Hdh-TK peptide, as described in further detail below.

Our phylogenetic analysis supports the closer relationship of Hdh-TKRs with the gastropod *Aplysia* TKR and other molluscan TKRs, forming a monophyletic clade, as would be expected for orthologous receptors. More specifically, the lophotrochozoan TKRs from mollusks and annelids are closely related, and they share a common ancestor that split before the divergence of the arthropod lineage TKRs and NTLRs. This phylogenic view coincides well with the previous results that the lophotrochozoan TKRs clustered in a distinct branch from the insect TKRs and their duplicated NTLRs (8, 11). Recently identified TKRs, a deorphanized *C. elegans* TKR and predicted *Asterias rubens* TKRs (46, 52), are positioned in different clades from the bilaterian TKR/NTLR superfamily, indicating their substantial diversifications during the evolution of the phyla Nematoda and Echinodermata. Further identification and characterization of TKRs in neglected phyla, such as echinoderms and cnidarians, can provide an opportunity for detailed exploration of the evolutionary processes of TKs and TKRs, and understanding of the possible pleiotropy of TK signaling and function.

Two abalone TKRs, Hdh-TKRL and Hdh-TKRS, are potently activated by diverse Hdh-TK peptides and the ligand-activated receptors activate PKC/Ca^2+^ and PKA/cAMP signal-transduction pathways. Like the *Octopus* and *Crassostrea* TKRs (11, 34), Hdh-TKRs trigger a Gq-PLC-PKC-dependent cascade and an increase in intracellular Ca^2+^ mobilization, supported by the robust luminescence responses of aequorin and SRE-Luc reporters. However, Hdh-TKRs exhibited a distinct response to the *Octopus* and *Crassostrea* TKRs, which are not involved in triggering intracellular cAMP accumulation in the PKA signaling pathway (11, 34). The interaction of TK peptides with a *C. elegans* TKR also did not show evidence for the induction of intracellular cAMP accumulation. In contrast, the *C. elegans* TK peptides induced a robust increase in intracellular Ca^2+^ in the TKR-expressing CHO cells (46). Nevertheless, most insect and vertebrate TKRs are activated by their endogenous TK peptides to stimulate the Gs/cAMP/PKA and Gq/Ca^2+^/PKC signaling pathways (1, 18, 53–55), suggesting that the signaling through bilaterian TKRs is diverse and complex. The insect TKRs can induce mitogen-activated protein kinase cascades through the cooperation of G proteins, leading to the phosphorylation of ERK1/2 (18, 54). In this study, we also demonstrated that ligand-stimulated Hdh-TKRs mediate the phosphorylation of ERK1/2 in HEK293 cells and that ERK1/2 phosphorylation is inhibited by PKA and PKC inhibitors, which is in line with the observations of intracellular cAMP accumulation and Ca^2+^ mobilization. Although inactivation of ERK1/2 by a PKC inhibitor was observed in both Hdh-TKRL- and Hdh-TKRS-transfected HEK293 cells, a PKA inhibitor strongly decreased the level of ERK1/2 phosphorylation in Hdh-TKRL-transfected cells, and to a lesser extent in the Hdh-TKRS-transfected cells. This result suggests that the longer ICD in Hdh-TKRL is a direct target for PKA phosphorylation. The shorter heptapeptide Hdh-TK1-1, which lacks three or four N-terminal residues, and other Hdh-TK peptides had equivalent potencies in the cAMP/PKA pathway *via* Hdh-TKRs, in contrast to the dramatic decrease in cAMP accumulation in an N-terminally truncated SP-activated human NK1R (42). However, another molluscan TK heptapeptides increased intracellular Ca^2+^ mobilization in the HEK293T cells transfected with *C. gigas* TKR at nanomolar concentrations (11). These observations suggest that the molluscan TK signaling systems are diverse and complex, similar to those of insect and vertebrate TK systems.

To identify the Hdh-TKR residues important for Hdh-TK binding, we performed docking simulations to predict the binding mode of Hdh-TK peptides. We estimated the critical interactions in the Hdh-TKR binding pocket. As the docking scores of five Hdh-TK peptides were consistent with the corresponding EC50 values in our reporter assay, these results suggest that the peptide activity relies on binding affinity to Hdh-TKRL. In addition, the docking models of Hdh-TK1-3 and TK2-2 with Hdh-TKRL suggest that the hydrophobic interactions mediated by aromatic amino acids in the peptides are the major driving force for binding. The docking models are similar to recent structures of SP-NK1R complexes showing that the SP residues are oriented along a hydrophobic interface formed by TMD6 (e.g., Trp261, Phe268) and TMD7 (e.g., Tyr287) of human NK1R (42, 56). Furthermore, Phe25 in the N-terminal ECD and Tyr92 in the ECL1 of human NK1R, required for the high-affinity binding of SP (51, 56, 57), are present at the corresponding positions in Hdh-TKRL. In contrast, the higher EC50 value of Hdh-TK1-2 is probably due to unfavorable contact of the hydrophobic residues in Hdh-TK1-2 with the polar residues (Asn119 and Asn234) in Hdh-TKRL, as the residues of the extracellular polar network are important for SP binding with NK1R (42, 56).

Transcripts of *Hdh-TK* precursors and *Hdh-TKR* are expressed in various tissues of Pacific abalone, but the transcripts were dominantly expressed in the neural ganglia compared to those in the peripheral tissues, including the gonads and the intestine. These results are generally consistent with expression profiles of the molluscan *Octopus* and *Crassostrea* prepro-*TKs* and *TKRs* (11, 34). In previous peptidome and transcriptome analyses, there was no different occurrence of Hdh-TK1 and Hdh-TK2 in pooled ganglia between immature and mature female abalone (37). Similarly, no dramatic difference in the expression of prepro-*TK* and *TKR* genes was noticed throughout the reproductive cycle in the visceral ganglia and the gonads of *C. gigas* (11). In vertebrates, however, central TK signaling is known to regulate reproductive activity by controlling the pituitary hormones (20, 58). The possible implications of TK signaling on the reproductive axis were also observed as higher expression levels of prepro-*TKs* and *TKRs* in the gonads and reproductive cells in chordate species (21, 59, 60). Interestingly, the prepro-*TK* transcript levels were higher in the brains of female honeybees (queens and foragers) than male honeybees (drones), implying that the expression of TK precursor is associated with sex or age/division of insect labor (7). Although we could not detect drastic differences in spatial *Hdh-TKR* transcripts between mature female and male abalone, transcript levels of the *Hdh-TK1* and *Hdh-TK2* precursors in the CG tended to be higher in females. This result suggests that the expression of the *Hdh-TK* precursor in the CG is regulated differently for each sex. However, little is known about invertebrate TK precursor expression in distinct ganglia and sexual differences and we are certainly interested in seeing a detailed picture of TK expression profiles in other species.

Of the many neuropeptides in model invertebrate species such as *Drosophila* sp. and *C. elegans*, TK peptides have emerged as the predominant suppressor in controlling the process of intestinal lipid metabolism. In gut endocrine cells of *Drosophila*, the TK peptide was shown to suppress lipogenesis *via* PKA/SREBP signaling in enterocytes of the midgut (10). *C. elegans* TK-like peptide (FLP-7) was also reported to act *via* the TK2 receptor (NPR-22) in the intestine and drive fat loss *via* an adipocyte TG lipase (61). In line with these studies, we revealed that transcript levels of the *Hdh-TK1* precursor increase in the neural ganglia when the intestinal TG content and transcript levels of *SREBP*, a transcriptional activator for lipid production (36), decrease in abalone starved for 3 weeks. Similarly, the *Hdh-TK1* precursor transcript levels increased over three-fold in the intestine and hepatopancreas of starved abalone, although there were no significant differences between fed and starved abalone. Given that the expression of the *Hdh-TK2* precursor was unchanged in the starved abalone, upregulation of the *Hdh-TK1* precursor induced by long-term starvation suggests that Hdh-TK1 peptides predominantly inhibit lipid production in starved abalone. Similar to the expression pattern of the *Hdh-TK1* precursor in abalone, *TK* precursor transcript levels in the visceral ganglia increased in four-week-starved oyster *C. gigas* (11). In CNS in mammals and *Drosophila*, the neural cells are highly active in lipid synthesis under the control of SREBP (62, 63). Thus, we speculate that Hdh-TK peptides regulate *de novo* synthesis of lipid in the ganglia *via* controlling SREBP expression to maintain neuronal functions such as dendrite expansion and the myelination of axons. Indeed, a single *in vivo* injection of the high dose of Hdh-TK peptides decreased the transcript levels of *SREBP* in the abalone CG, although we were unable to obtain direct evidence of increased *Hdh-SREBP* transcript levels in the CG following administration of a single dose of *Hdh-TK* siRNA. On the contrary, intestinal *Hdh-SREBP* levels were increased following *Hdh-TK2* siRNA injection, suggesting that TK signaling and SREBP-mediated changes in lipid metabolism are likely tissue-specific in mollusks. Other than the expression pattern of TK precursors, it is largely unknown whether molluscan TK signaling controls food intake. Although the injection of the lower dose of Hdh-TK1 peptides showed an orexigenic effect in Pacific abalone, no consistent effects were observed by a higher dose of Hdh-TK peptides, including Hdh-TK2. In the German cockroach, TK injections increased food content in the foregut at 5 h post-injection (64). Conversely, TK peptides may have anorexigenic actions in vertebrates, although direct evidence for a role of TK peptides in regulating food intake is lacking (65). In the future, detailed studies are necessary to reveal whether molluscan TK signaling affects food intake, as in other species. In addition, the downregulation of *Hdh-TKR* expression in the ganglia by long-term starvation is in contrast to the unchanged *TKR* expression profiles in *C. gigas* and *D. melanogaster* that were starved for short or long periods (11, 23). However, it is noteworthy that intestinal *Hdh-TKR* expression was also downregulated by starvation, although no significant difference was observed between fed and starved abalone. More studies examining the TKR signaling for different nutritional status and starvation periods are needed to fully unravel their complex roles in the CNS and peripheral tissues.

In summary, we have identified two Hdh-TK precursors and functionally characterized two Hdh-TKR isoforms in the Pacific abalone to better understand the molluscan TK signaling system. Five Hdh-TK peptides derived from Hdh-TK precursors could activate the cAMP/PKA and Ca^2+^/PKC signaling pathways through Hdh-TKRs. The *Hdh-TK* precursor and *Hdh-TKR* genes are globally expressed in diverse tissues. The transcript levels of the *Hdh-TK* precursor and *Hdh-TKR* are relatively higher in the neural ganglia than in the peripheral tissues. The abalone TK signaling pathway is most likely involved in the lipid synthesis process in the neural ganglia, intestine, and hepatopancreas by controlling SREBP.

## Data availability statement

The datasets presented in this study can be found in online repositories. The names of the repository/repositories and accession number(s) can be found in the article/**Supplementary Material**.

## Author contributions

SL and YS designed the study. SL, J-MP, and MK performed bioinformatic analysis and biological experiments. KP constructed *in silico* receptor–peptide binding modeling. All authors contributed to the article and approved the submitted version.

## Funding

This work was supported by the National Research Foundation of Korea (NRF) grant funded by the Korea government (2020R1A2C2009872) and Korea Institute of Science and Technology intramural research grant and Gangneung-Wonju National University.

## Conflict of interest

The authors declare that the research was conducted in the absence of any commercial or financial relationships that could be construed as a potential conflict of interest.

## Publisher’s note

All claims expressed in this article are solely those of the authors and do not necessarily represent those of their affiliated organizations, or those of the publisher, the editors and the reviewers. Any product that may be evaluated in this article, or claim that may be made by its manufacturer, is not guaranteed or endorsed by the publisher.
