# Supplementary material for: Multiple tachykinins and their receptors characterized in the gastropod mollusk Pacific abalone: Expression, signaling cascades, and potential role in regulating lipid metabolism
Source: Front Endocrinol (Lausanne). 2022 Sep 12;13:994863. doi: 10.3389/fendo.2022.994863 (PMC9521575; doi:10.3389/fendo.2022.994863)
Supplement: Supplementary file 1 [file DataSheet_1.pdf]

Supplementary Figures

1 CGAAAGGTTTTGAGTTGGCATACGCCACATTAGTCTTGTCGAATTGTCGATATCACTG 58
59 CGATTGCAGCCAACACAAGCACTGTGACCACGCCCCCTGCGTGTCTCCTAGCCTTGCGTG 118
119 GGAAAACACCTCAATAGCGTGTGTGATCGATGCCTCTCTGAAAGCATTGTATAAAGACTG 178
179 TCACTAGTACAGAATCACCGTCAGACTCGAAGGAACGTCTGTCCACTCATCAAGGGAACC 238
239 GAGACAACTACCGGCTAACGCCACATTACCCCTCAACGTTTAAAGCAACCTTCAACAAAC 298
299 ATGTCCTCAAGCCAAGGTGTGATATTTGTACTGGCAGGATTTGCTCTTCTCGCATTGGTC 358
1 M S S S Q G V I F V L A G F A L L A L V 20
359 AACGCAGAGGCATTAGATGATAACACAGCTGCAAGTCTATACAAGTTATTACAACCAGCC 418
21 N A E A L D D N T A A S L Y K L L Q P A 40
419 TATCAATCAACAGTAGCAGAGAAGCGGGAGAGTGACCTCAACCTTGCCTTCGATGCCCCA 478
41 Y Q S T V A E K R E S D L N L A F D A P 60
479 CCCGATCCCAACTGGAGTGCGGGTCCTTCAACCTACGGCTTCGGGTCCCTACTGAGACGC 538
61 P D P N W S A G P S T Y G F G S L L R R 80
539 GCGCCCGCGGGCTACAGGAGGAACCTCATCGAAGACAAGCGTTTTGGATACGTTGGCAGC 598
81 A P A G Y R R N L I E D K R **F G Y V G S** 100
599 AGAGGAAAGAGGACAGAACTAGGATTCGGATATGTTGGAAGCAGAGGAAAACGCACGAAT 658
101 **R** G K R **T E L G F G Y V G S R** G K R T N 120
659 CATGCCACCGCCTTTGTTACGTTAAGAGATCTTTTGGATATGTACGAAGAGCGAGCTAGA 718
121 H A T A F V T L R D L L D M Y E E R A R 140
719 GGATTACCCTTCCGAGCATCTGATCTCAACAGCCTCAGCCAAGATTCCGCTTTGCTCAAC 778
141 G L P F R A S D L N S L S Q D S A L L N 160
779 AGTAAACGACAACCACACTTCGGATTCCACGGTGTGAGAGGTTGA 823
161 S K R **Q P H F G F H G V R** G * 174

824 ACATACCCACGCCTAGTGTGAGTGGAACTTTGATGCTGAAACTGACAACATACCTGTAAA 883
884 CGGAATGCAGTGTTTTGACTGAAGGCGGAAATTCAAGTGGAACTGCTCTTACCACAATGC 943
944 GGGATATTACCTTACAACAGAGGACACGTGACAAAAAAATCAACATTTTTTTCAAGACTC 1003
1004 GTGACAAGATAAACAGTATCCTTGTGTGACATTCATTATAATATGAAACGTAGGACCGTC 1063
1064 AGCCGTACAAACATTCTAGGTTTACTCTCATTTATTGTAAATAAAATCCGTGAAAGAAGA 1123
1124 AATGTTTTATTGGTGCATCACGTGAAATGATGCATATGGCACGTGACAAACAAGCACTTC 1183
1184 CGTCCGGGGATACAGATAAATTCCTTCAACTAACTGACAAGAAAGAACTATCATGATCAT 1243
1244 GCTGGTGTCCACCTGGGAGGGAGAGAGCCACTTAACCAAGGAATTCAACTTCATGAAGTG 1303
1304 TGATATTTGAGAATAGACAATTGTACAGAGATTATGATGTTCATTTCCTTTTTCATTCTT 1363
1364 CGTTTGTATCACAATGTAGGTTGTCTGATAGAGATTGTCCATTATATCAATTCAGAGTTA 1423
1424 TACATTCCCCTATTTTCATAATGATTTGTTTGTTATTTCGTTGCTTGTTAGAATTCATTT 1483
1484 TGTATGAAAATATCAGAAGCATTGGACATGTTGTTTATTTGTTCACTTTATTTAATGCTA 1543
1544 GATTCATGAATACACACCATCAGATTTTGCCTTTGAAACGTAACGCCTGATGCCAATATT 1603
1604 ATATATACTGCACATAAAATAGGTATGGAATTACGAATATTACTTCGATTCTGGCTTGTT 1663
1664 TCACCCCTTAAACATATTTATGTGGCATTTCTGGCGGTGTATATTCTTTTTAGAGCTGCG 1723
1724 GGTGATTATACGCTTGTACATTTCACTATATATCTCGTGATATATCCGTTTCTTCTATTT 1783
1784 TTGTCTCTGCAAATAGAAAATATTTTAAAATAAATTTGTCTTCAAAACTAATCGTCCGTG 1843
1844 CTCCTCTGTGGTAAATAAAAATCTAAGAGTTGCA 1877

Supplementary Figure 1A

1 CGGGTTTCCC 10
11 ACTCGCCCATAATCCTATATAAAACAGGGCAGCCGACTGAATTACAGTCAGCACAACCCC 70
71 AGCTTCGAGTTCGATCAGCATTATCATTGGAGAGGTGATCTGTGAGACCACAGATCGAAC 130
131 TGGCTGGATACTCTAATAGTAAGCGAAGGTGACACGAATCTCTCCCAACAGAGGGCGCAT 190
191 CGGGTCTTTCTATTCCGAACTTTGCGTCAGGACAGTAGCACAAAATTAGCGGATAAGCT 250
251 TAACTTGAGGAGCGAGTTCAAATCTATTTAACGGTTGTCGAGGGCCAGCCCCAGTTGGAG 310
311 ATGTGGAAAGTGTCGACTCTGTGTATTGTGCTTGTGTGCGGGTTTGTGGGAACACAGGCT 370
1 M W K V S T L C I V L V C G F V G T Q A 20
371 GAGAAAGAAACACACGACGAATCTGTGAAGCACAAAGGTAAGATCAACTTTACAGAAGGC 430
21 E K E T H D E S V K H K G K I N F T E G 40
431 AGCAGCTTAACGTCAAATCCTTCCCCTACCCATAACACTCAGGAGGACCCCGATGCCTTT 490
41 S S L T S N P S P T H N T Q E D P D A F 60
491 ATGGACTCTCTTCTAGGGTACAGGATACCCTTGAGCACGGATGATAATGAACTAAAGAAA 550
61 M D S L L G Y R I P L S T D D N E L K K 80
551 CGCGGCAGGCATTTTGGGTTCGTCGGGAGTAGAGGACGACGCGGGAAGACGTCTGTAAAT 610
81 R **G R H F G F V G S R** G R R G K T S V N 100
611 TCCTTTTCCGAGGACCTATTCCCTAAACGACGCCCGATGAGCAACAGTGCACGTCTCCGG 670
101 S F S E D L F P K R R P M S N S A R L R 120
671 ATGCTCCTTGAGAACCGAGACCAAGGGTTGTTTGCCAAGGAGAAACGAAAGCCTCACTTT 730
121 M L L E N R D Q G L F A K E K R **K P H F** 140
731 GGGTTCCATGGCTCCAGAGGTTAA 754
141 **G F H G S R** G * 147
755 GGGGCTGACCGTGAATGAATGGAGGGAACCAACTGGAATGAGTTTGGAGAAAACAACCTT 814
815 GAAGGCATTCTTGTAAACTACTCTATGCACCCACGTATCTGCTTGAGAACACCGCATCTT 874
875 TATACGGCATCCCTGTCTCCACGTGGTGACACGGCACACCATCGCTGTAGACACGACACA 934
935 GTTTAATGTTAACAAGTACAAGAAGTCTGTTCTCACGCCATTCGTCGTTGCTACAAAAGC 994
995 GATAGATGTTTATTGTTAGCCTTTGGGTATCCATGGACTTTTACAATAAAATGTTTCTTT 1054
1055 ATTAACTATCATATACATTTCTCTCATCAAGAATTCAATCAAGGGTTGGTCTTCGAGACA 1114
1115 TGTGAAATAAACTTTTAACTGCAATGGATCAATGAAATTAAAGGTATACACAGAGTGGCA 1174
1175 AATATATTTACTTTTACAAAATAAGTGAGGGGTCACATGGAGTACTTGTCAAAGTGGTTG 1234
1235 AAATTATATACACAACATAATGACACTATAACAAGCCGCTAAGAATGCGCGATCCGTTTT 1294
1295 GATGACTGTCGATGATATAATTCCTAGAGTATCACCACATGCTTATATTATAGTGGGAAT 1354
1355 CGTACAACGCGTCTGCTTTGAGCAGATAGTTCTCATTACTATGGAAAGGGATGTGTTGCT 1414
1415 TTTATATTCGATTAATCCTGTTTTCCCAAACTTATCTTAATTACAAATCACAAACAAGAA 1474
1475 GCCATCAATCAGGATGACACCATTCAGAACACAGAGACATCGTTTGGGGTACGTTACACT 1534
1535 TCAAAGTTTGAGATAACGGGAATTGGCAGTATCTTGAAAAGGGGTATAGAAAATGTTACA 1594
1595 TTTCATCTAAGGTGTGTGTTTAACAAAGTCTTTGTCCTTAAGATTCAGCCAAAATGAACA 1654
1655 CTTTGTAACGGTAGTTTTGTATAGCCACAACTCGGAAACCATTATGGTTTTTGTACGATT 1714
1715 TCCATTATTTGTCGCGTCATTTCCCACATTGTCACGATATATTTGGAGCATTTCAACTCA 1774
1775 CTCAATAAGAAACTAGCACTGTTTTCTCTTCGCCGTCATTCTTTGAATTGAACAGTTGAT 1834
1835 GCTAAGTAATCAATTCGTTATTGTCTATAGAGTAGAACATTTCGTCTTTGGGACATTAAG 1894
1895 CCTGGTTATTTCATTAAAAAAATATGTACCATGAATTTTAAGTATTCCGCAATGTGGAGG 1954
1955 GTTAGTACCCTCGGATGGAACATGGAAGTCTTCGAGGCAATGTTAGAATATGATCTGGTT 2014
2015 TGGTTCAAAGACTCAAAGGTGTCCGACATTGATCTGAATTCACAGAATAAAGCATGGCTT 2074
2075 GATG 2078

Supplementary Figure 1B

***Hdh*-TK1-1**	**--------------FGYVGSRG**
***Hdh*-TK1-2**	**----------TELGFGYVGSRG**
***Hdh*-TK1-3**	**-------QPH----FGFHGVRG**
***Hdh*-TK2-1**	**-----------GRHFGFVGSRG**
***Hdh*-TK2-2**	**-------KPH----FGFHGSRG**
C.gigas-TK1	--------------FGFAPMRG
C.gigas-TK2	--------------ARFFGLRG
C.gigas-TK3	--------------FRFTALRG
O.vulgaris-TK1	-----------VNPYSFQGTRG
O.vulgaris-TK2	-----------LNANSFMGSRG
O.vulgaris-TK3	----------TVSANAFLGSRG
O.vulgaris-TK4	-----------SDALAFVPTRG
O.vulgaris-TK5	-----------MNSLSFGPPKG
O.vulgaris-TK6	-----------YSPLDFIGSRG
O.vulgaris-TK7	----------ASLHNTFIPSRG
U.unicinctus-TK1	-----------LRQSQFVGARG
U.unicinctus-TK2	-----------AAGMGFFGARG
U.unicinctus-TK3	-----------AAPSGFFGARG
U.unicinctus-TK4	-----------AAYSGFFGARG
U.unicinctus-TK5	-----------APSMGFFGARG
U.unicinctus-TK6	-----------APHMRFYGSRG
U.unicinctus-TK7	-----------APKMGFFGARG
C.elegans-TK1	SGPSSASEGEAYAFPGLRGLRG
C.elegans-TK2	----DPTYHKRVPMMSLKGLRG
C.elegans-TK3	-----------VPMMSLKGLRG
D.melanogaster-TK1	-----------APTSSFIGMRG
D.melanogaster-TK2	------------APLAFVGLRG
D.melanogaster-TK3	------------APTGFTGMRG
D.melanogaster-TK4	-----------APVNSFVGMRG
D.melanogaster-TK5	------------APNGFLGMRG
A.aegypti-TK1	------------APSGFLGLRG
A.aegypti-TK2	------------VPSGFTGMRG
A.aegypti-TK3	------------APSGFLGMRG
A.aegypti-TK4	------------VPNGFLGVRG
T.castaneum-TK1	------------APSGFTGVRG
T.castaneum-TK2	------------APSGFMGMRG
T.castaneum-TK3	------------APMGFMGMRG
T.castaneum-TK4	------------APSGFFGMRG
T.castaneum-TK5	----------MPRQAGFFGMRG
T.castaneum-TK6	--------YPYQFRGKFVGVRG
B.mori-TK1	------------IPQGFLGMRG
B.mori-TK2	------------APLGFTGVRG
B.mori-TK3	----------AANMHQFYGVRG
B.mori-TK4	--------YDLSIRGKFIGVRG
B.mori-TK5	------------GQMGFFGMRG
L.migratoria-TK1	------------GPSGFYGVRG
L.migratoria-TK2	-----------APLSGFYGVRG
L.migratoria-TK3	-----------APQAGFYGVRG
L.migratoria-TK4	-----------APSLGFHGVRG
L.migratoria-TK5	-----------APMRGFQSVRG
L.migratoria-TK6	------------ALKGFFGTRG
L.migratoria-TK7	-----------APSAGFHGVRG
L.migratoria-TK8	------------APVGFYGTRG
Exocrine *O.vulgaris*-TK1	---------KPPSSSEFIGLMG
Exocrine *O.vulgaris*-TK2	---------KPPSSSEFVGLMG
Exocrine *O.vulgaris*-TK3	---------DPPSDDEFVSLMG
Exocrine *A.aegypti*-TK1	-----------NTGDKFYGLMG
Exocrine *A.aegypti*-TK2	-----------DTGDKFYGLMG
Exocrine *E.moschata*-TK	----------QPSKDAFIGLMG
H.sapiens-SP	----------RPKPQQFFGLMG
H.sapiens-NKA	-----------HKTDSFVGLMG
H.sapiens-NKB	-----------DMHDFFVGLMG

Supplementary Figure 2

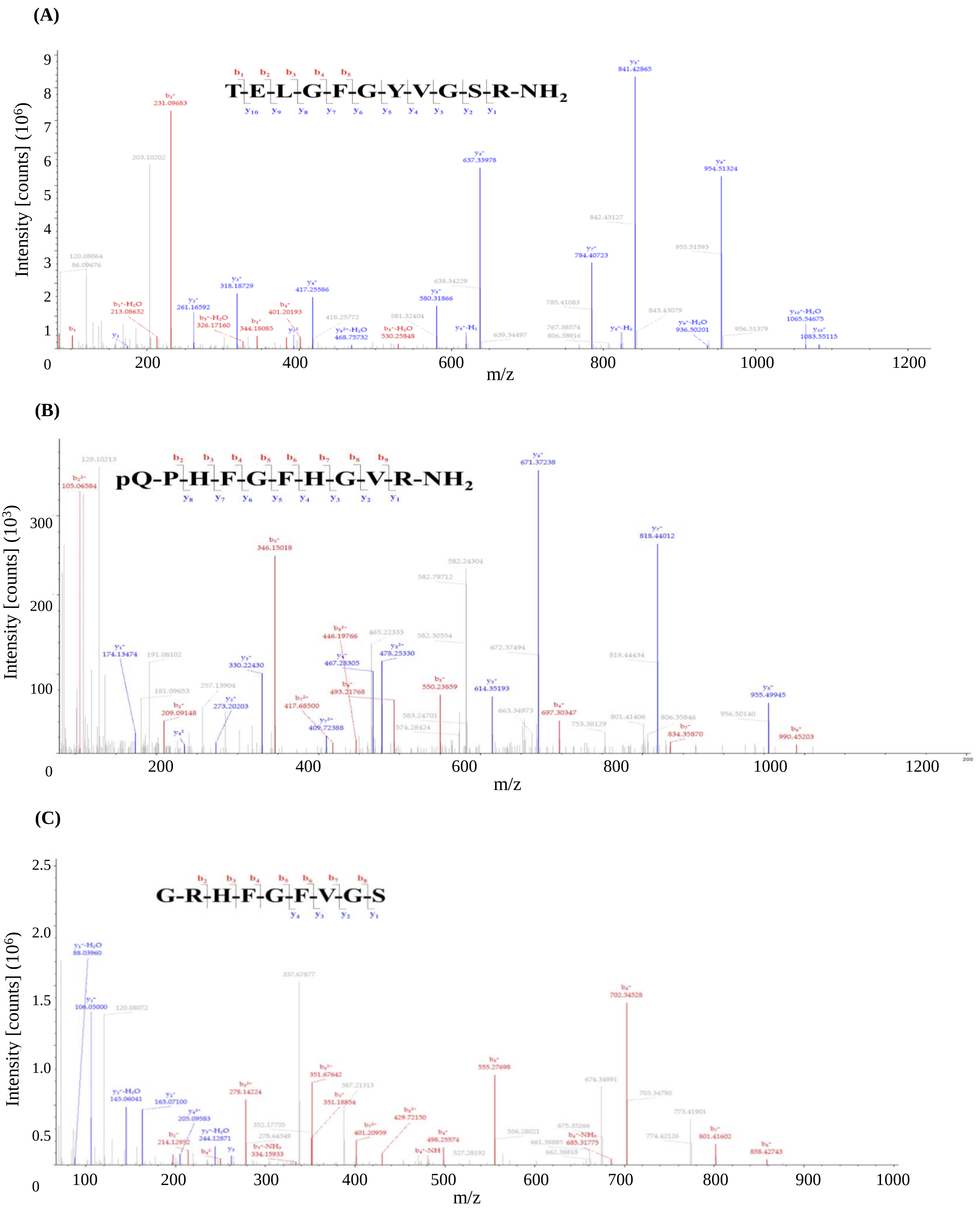

Supplementary Figure 3

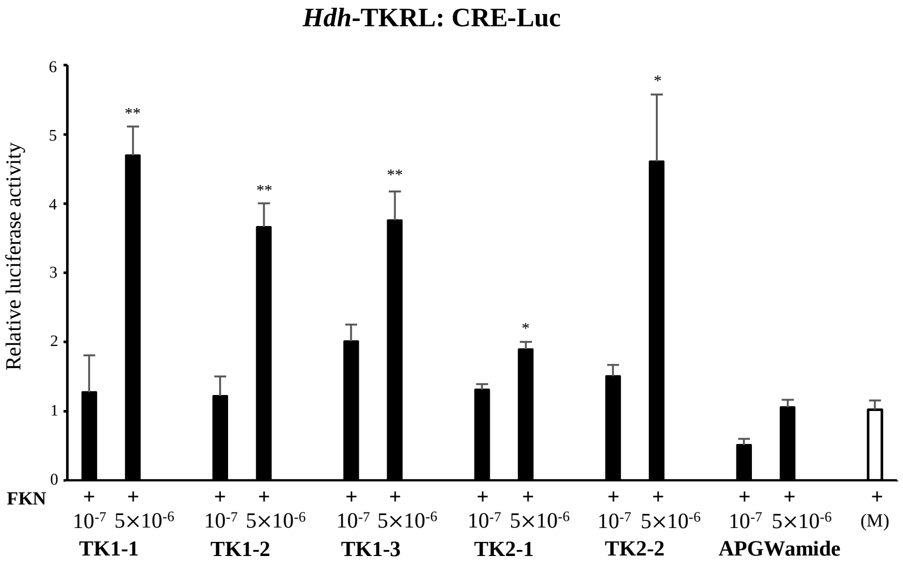

Supplementary Figure 4

(A)

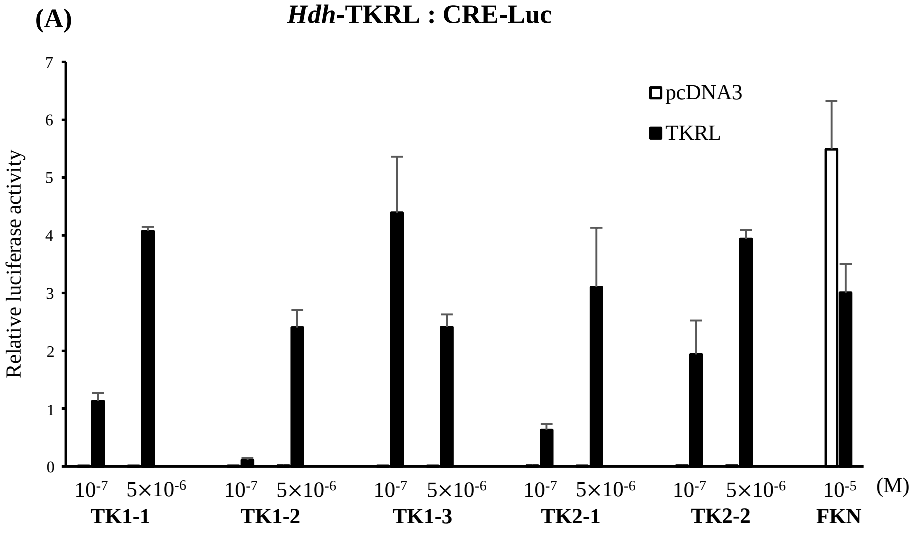

(B)

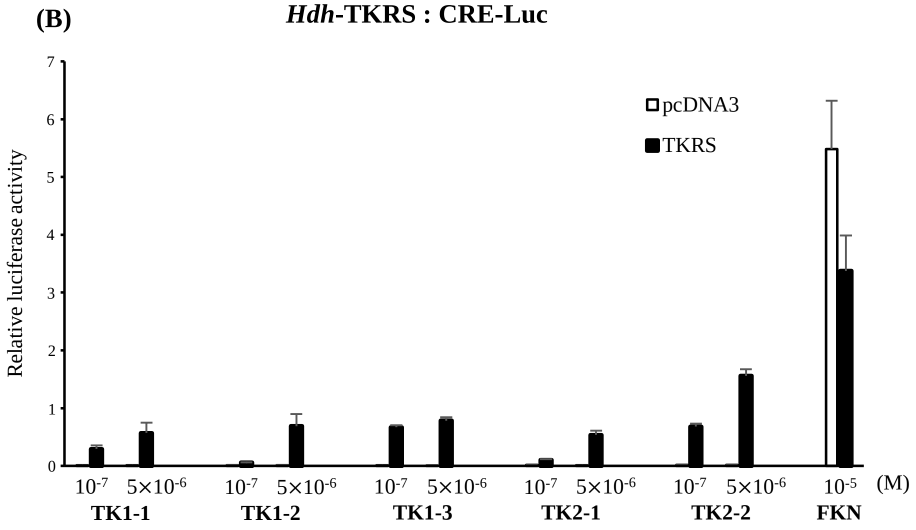

Supplementary Figure 5

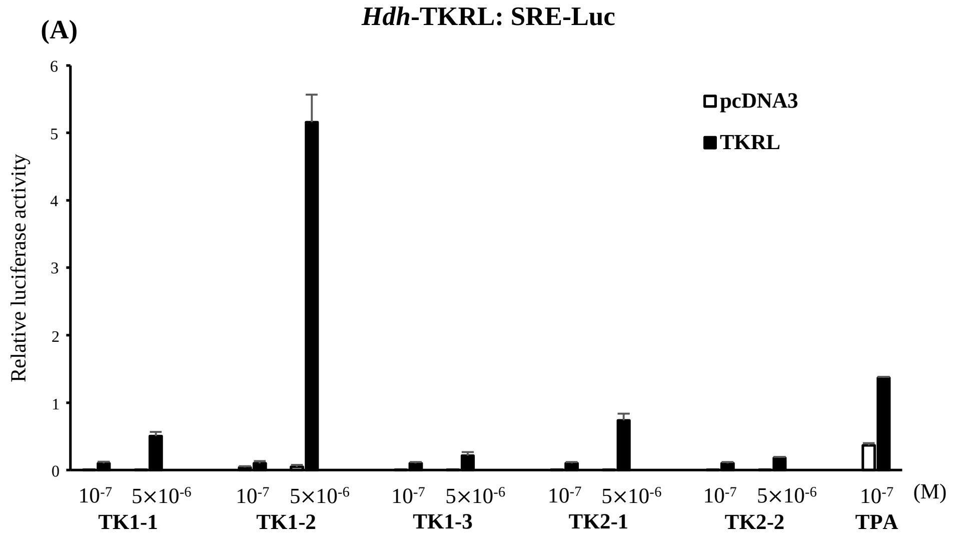

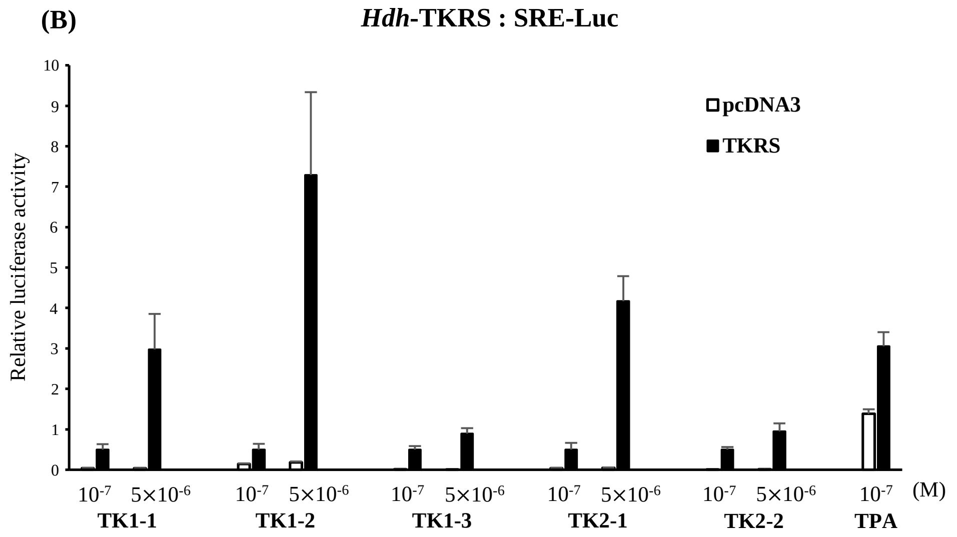

Supplementary Figure 6

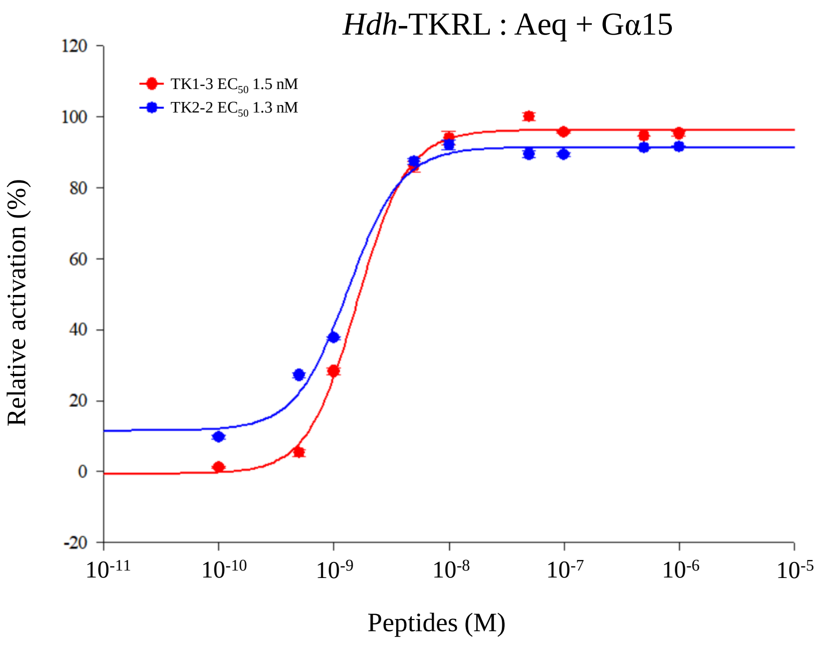

Supplementary Figure 7

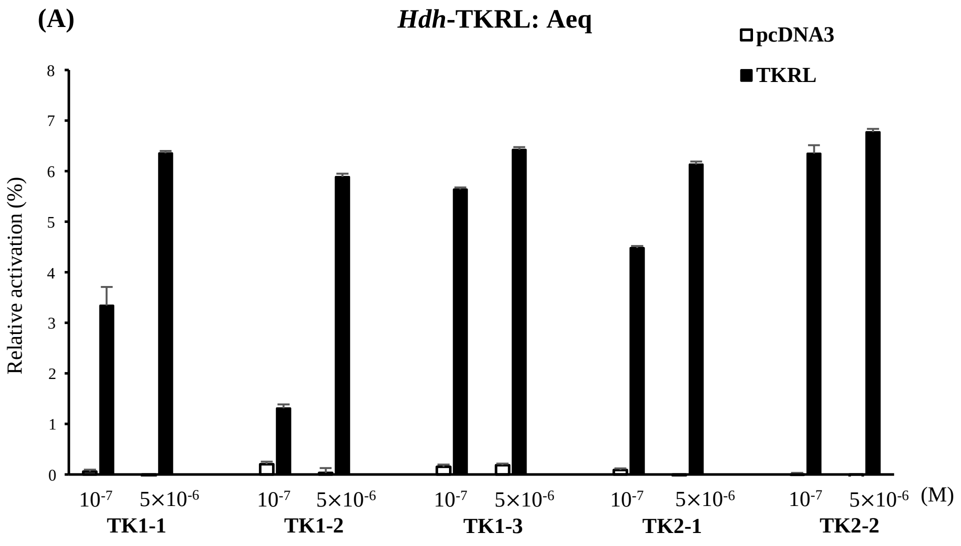

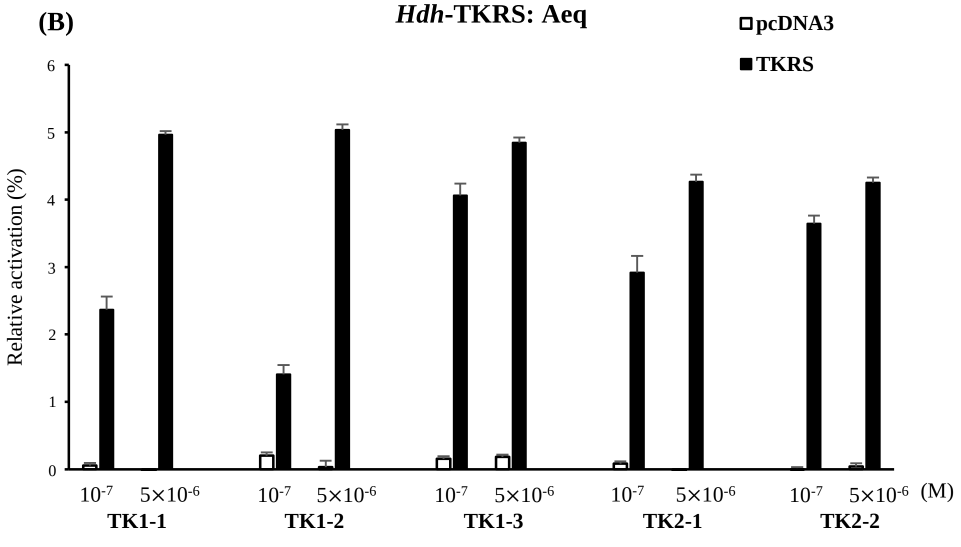

Supplementary Figure 8

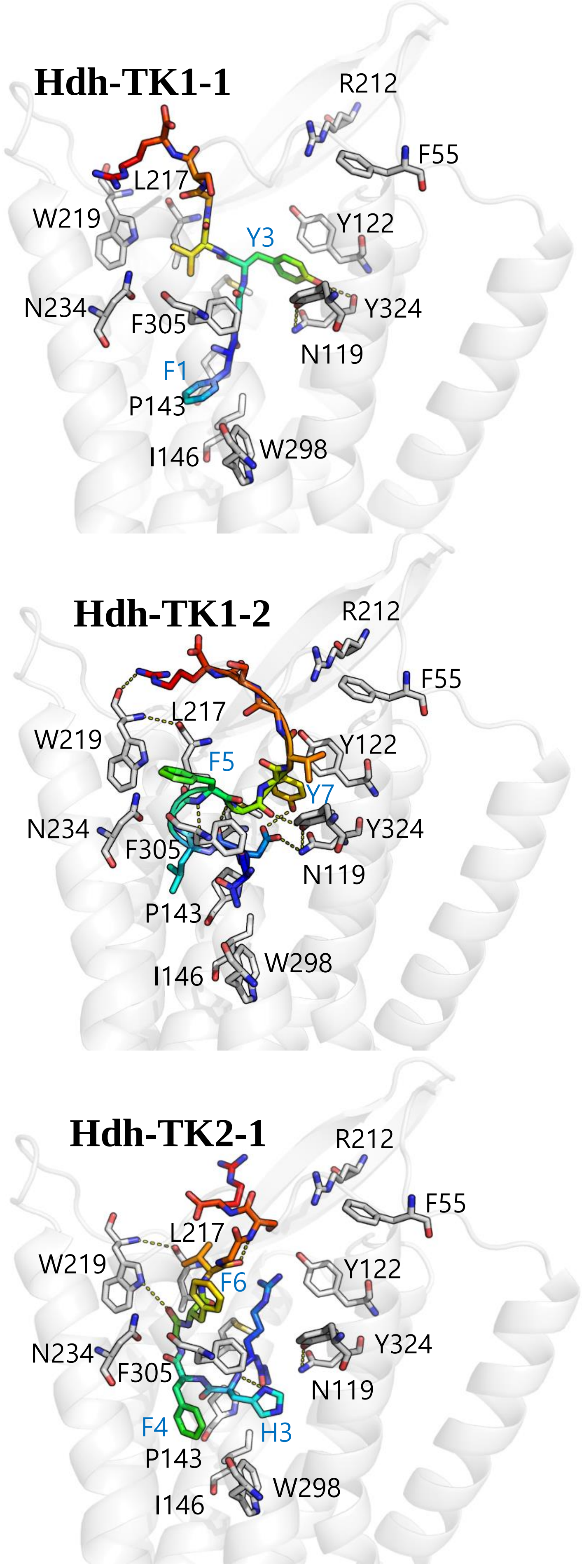

Supplementary Figure 9

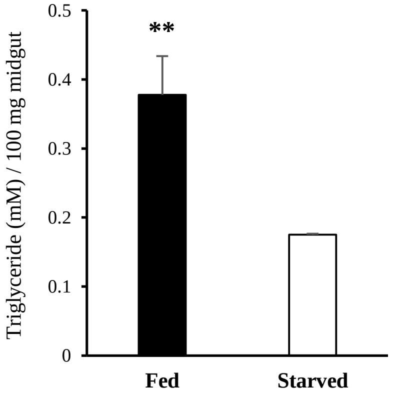

Supplementary Figure 10

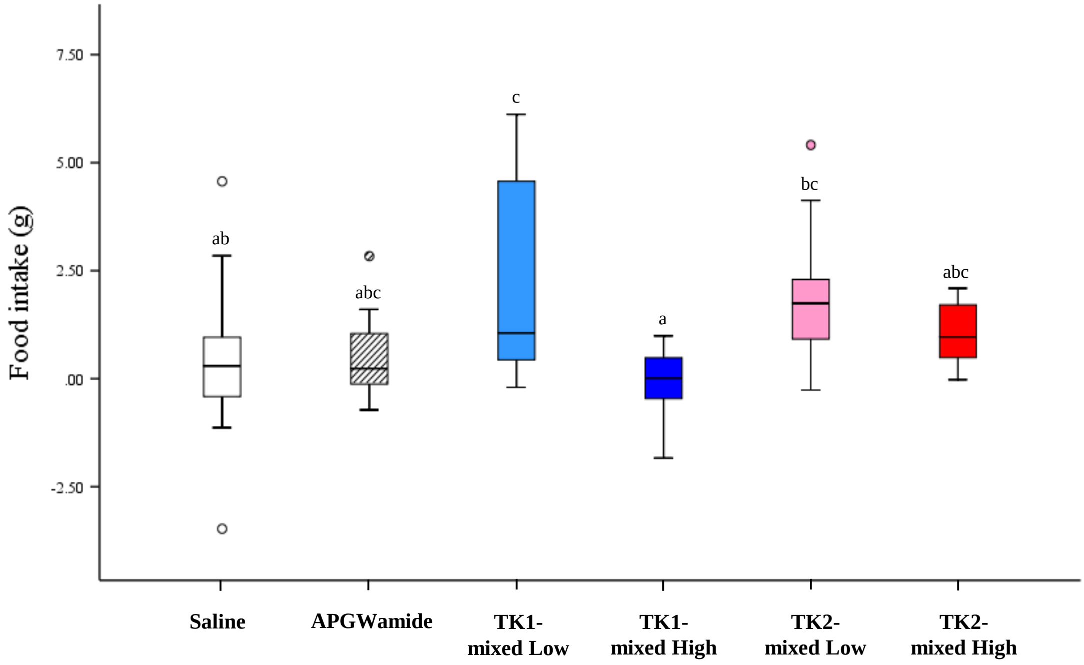

Supplementary Figure 11

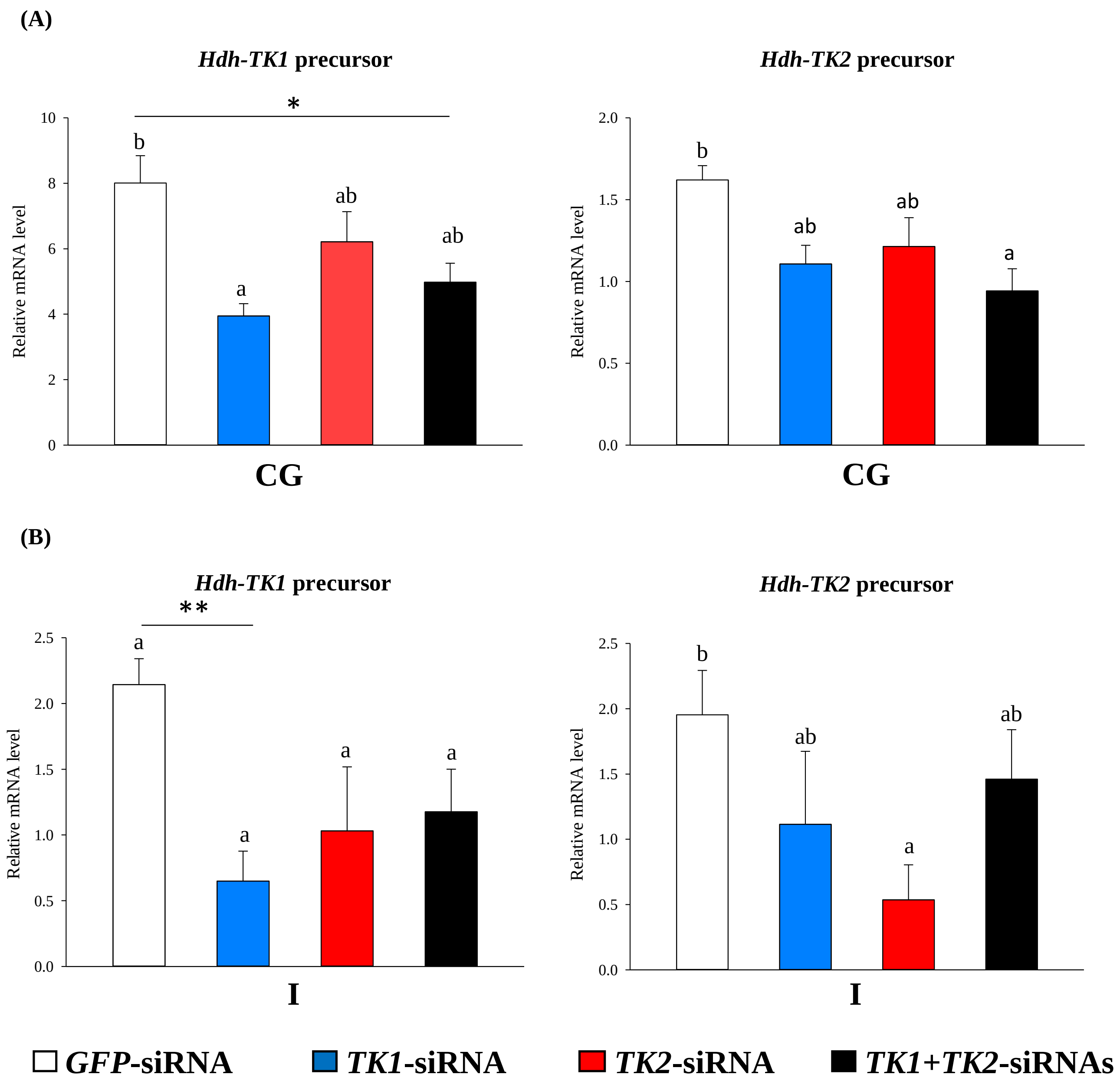

Supplementary Figure 12

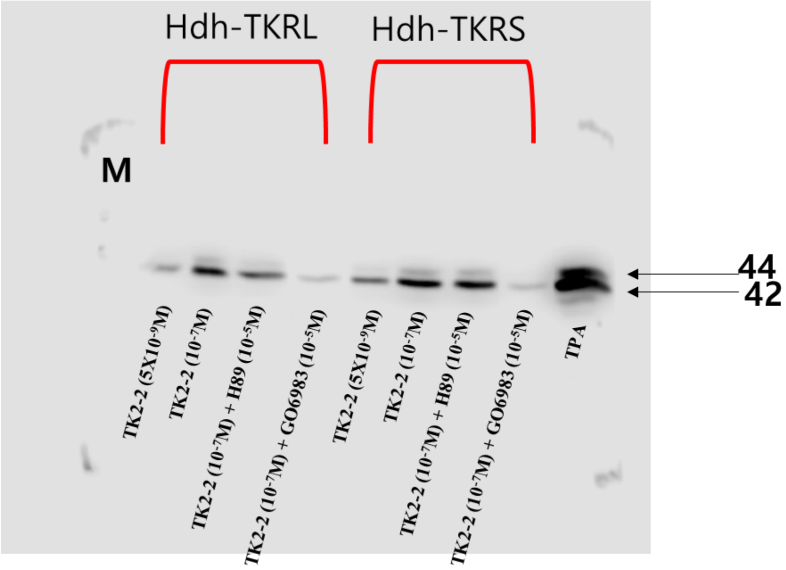

Phospho ERK

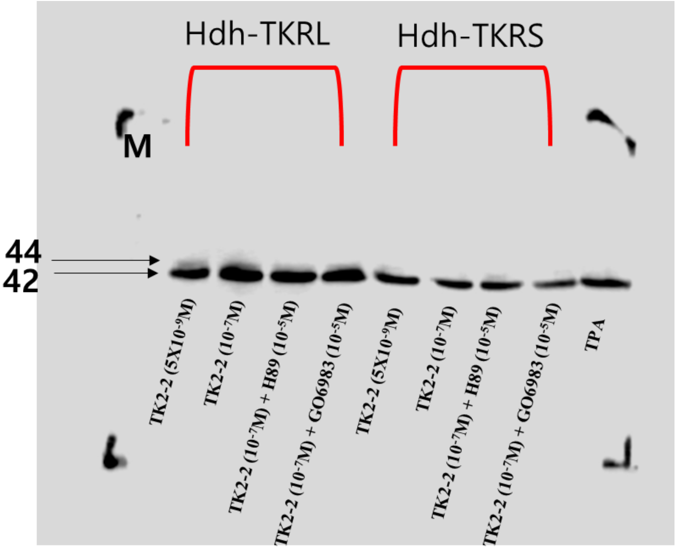

Total ERK

Supplementary Figure 13

Supplementary Figure Legends

Supplementary Figure 1. The full-length nucleotide and predicted amino acid sequences of Hdh-TK1 and Hdh-TK2 precursors. (A) Hdh-TK1 precursor cDNA contains 1877 bp nucleotides and encodes 174 amino acids. (B) Hdh-TK2 precursor cDNA contains 2078 bp nucleotides and encodes 147 amino acids. The predicted TK mature peptides are shown in bold letters. The signal peptide sequence and dibasic cleavage site residues are underlined with solid and dashed lines, respectively. An asterisk denotes the stop codon. The nucleotide sequences for Hdh-TK1 and Hdh-TK2 precursors have been deposited in the NCBI GenBank database (Hdh-TK1, MZ197811; Hdh-TK2, MZ197812).

Supplementary Figure 2. Multiple sequence alignment of TK mature peptides in bilaterian species. Black and gray shaded amino acids represent identical and similar residues, respectively. Supplementary Table 1 provides information for sequences.

Supplementary Figure 3. MS/MS spectra acquired by the nano-LC-MS/MS system for (A) TELGFGYVGSR-NH_2 (Hdh-TK1-2, m/z 1184.606), (B) pQPHFGFHGVR-NH_2 (Hdh-TK1-3, m/z 1163.586), and (C) GRHFGFVGS (Hdh-TK2-1, m/z 963.4795). The presence of b- and y-ions was indicated by horizontal lines above (b-ions) or below (y-ions) the corresponding amino acid residues. Recorded ion signals are labeled as the monoisotopic masses $[M+H]^+$.

Supplementary Figure 4. Effect of Hdh-TK peptides on forskolin (FKN)-stimulated CRE-Luc reporter activities in Hdh-TKRL-expressing HEK293 cells. The relative luciferase activities were determined in response to 10^{-5} M of FKN along with TK peptides or APGWamide (10^{-7}, 5×10^{-6} M). All data represent the mean ± SEM (n = 4). Statistical significance was tested by Student's t-test. *$p < 0.05$, **$p < 0.01$.

Supplementary Figure 5. Effect of Hdh-TK peptides on CRE-Luc reporter activity in Hdh-TKR-expressing HEK293 cells. Intracellular cAMP accumulation was measured by CRE-Luc reporter activities in (A) Hdh-TKRL-, (B) Hdh-TKRS-, or maternal plasmid pcDNA3-transfected HEK293 cells. The relative luciferase activities were determined in response to TK peptides (10^{-7}, 5×10^{-6} M) or forskolin (FKN, 10^{-5} M). All data represent the mean ± SEM (n = 3).

Supplementary Figure 6. Effect of Hdh-TK peptides on SRE-Luc reporter activity in Hdh-TKR-expressing HEK293 cells. SRE-Luc reporter activities were measured in (A) Hdh-TKRL-, (B) Hdh-TKRS-, or maternal plasmid pcDNA3-transfected HEK293 cells. The relative luciferase activities were determined in response to TK peptides (10^{-7}, 5×10^{-6} M) or 12-O-tetradecanoylphorbol-13-acetate (TPA, 10^{-7} M). All data represent the mean ± SEM (n = 3).

Supplementary Figure 7. Dose-response curves for intracellular Ca^{2+} mobilization in Hdh-TKRL-, Gα15-, and aequorin-expressing CHO-K1 cells. Luminescence was plotted relative to the maximal response achieved when TK peptides (10^{-6} M) were applied to the CHO-K1 cells. Data represent the mean ± SEM (n = 4).

Supplementary Figure 8. Effect of Hdh-TK peptides on intracellular Ca^{2+} mobilization in Hdh-TKR- and aequorin-expressing CHO-K1 cells. Aequorin reporter activities were measured in (A) Hdh-TKRL-, (B) Hdh-TKRS-, or maternal plasmid pcDNA3-transfected CHO-K1 cells.

The relative luminescence activities were determined in response to TK peptides (10^{-7}, 5×10^{-6} M). All data represent the mean ± SEM (n = 3).

Supplementary Figure 9. *In silico* docking model of the Hdh-TK-TKR complex. The figure scheme is identical to Figure 8. The bound Hdh-TK1-1/TK1-2/TK2-1 peptides are represented by color gradient from blue (N-terminus) to red (C-terminus). The receptor backbone and binding site residues are shown in white. Yellow dotted lines represent the hydrogen bonds. The hydrogen bonds are represented by dotted lines. The receptor backbone and binding site residues are shown in white and the aromatic residues in each peptide are indicated in blue.

Supplementary Figure 10. Triglyceride concentration in the midgut of fed and starved abalone for 3 weeks. Data represent the mean ± SEM (n = 6). Statistical significance was tested by the student's t-test (**p < 0.01).

Supplementary Figure 11. Effect of Hdh-TK peptides on kelp consumption in abalone. At 24 h after injection of Hdh-TK1 and TK-2 peptide mixtures (low dose, 0.25 µg/g BW; high dose, 2.5 µg/g BW) along with an equal volume of saline and APGWamide (2.5 µg/g BW), food intake was calculated. All data represent the mean ± SEM (n = 8). Different letters on the bars indicate statistically significant differences (p < 0.05).

Supplementary Figure 12. Relative expression levels of *Hdh-TK1* and *-TK2 precursors* in the cerebral ganglion (A) and intestine (B) after administration of *Hdh-TK1* and/or *Hdh-TK2* specific siRNAs (50 µg/ind.). As a control, an equal amount of *GFP*-specific siRNA was administrated. At 44 h post-injection, tissues were sampled and relative *Hdh-SREBP* transcript levels were measured by real-time quantitative PCR. All data are presented as the mean ± SEM (n = 7 or 8). Different letters on the bars indicate statistically significant differences (p < 0.05). Statistical significance was tested by Student's t-test, *p < 0.05, **p < 0.01. Abbreviations: CG, cerebral ganglion; I, intestine.

Supplementary Figure 13. Full-length blots of phosphorylated and total ERK1/2 in Figure 7.

Supplementary Table 1. Sources and accession numbers of the TK precursors and peptides used for schematic representations and sequence alignments, as shown in Figure 1 and Supplementary Figure 2.

**Sequence Name**	**Species**	**Accession number**	**Reference**
Hdh-TK1	*Haliotis discus hannai*	MZ197811	This study
Hdh-TK2	*Haliotis discus hannai*	MZ197812	
C.gigas-TK (*Cg*-TK)	*Crassostrea gigas*	-	1
O.vulgaris-TK (*Ov*-TK)	*Octopus vulgaris*	AB037112.1	
U.unicinctus-TK (*Uu*-TK)	*Urechis unicincus*	AB019537.1	
D.melanogaster-TK (*Dm*-TK)	*Drosophila melanogaster*	AC020418	2
A.aegypti-TK (*Aa*-TK)	*Aedes aegypti*		3
C.elegans-TK (*Ce*-TK)	*Caenorhabditis elegans*	NM001136435.3	
D.melanogaster-natalisin (*Dm*-NTL)	*Drosophila melanogaster*	CG34388	
T.castaneum-TK	*Tribolium castaneum*	KYB25860.1	
B.mori-TK	*Bombyx mori*	AB298929.1	4
L.migratoria-TK	*Locusta migratoria*	KP895539.1	5 – 7
Exocrine *Ov*-TK1	*Octopus vulgaris*	AB085916.1	8
Exocrine *Ov*-TK2	*Octopus vulgaris*	AB085917.1	8
Exocrine *Ov*-TK3	*Octopus vulgaris*	-	9
Exocrine *Aa*-TK1 (sialokinin-1)	*Aedes aegypti*	AF108101.1	10
Exocrine *Aa*-TK2 (sialokinin-2)	*Aedes aegypti*	-	10
Exocrine *E.moschata*-TK (eledoisin)	*Eledone moschata*	-	11
Hs-Tac1α (SP)	*Homo sapiens*	NP_054702.1	
Hs-Tac1β (SP, NKA)	*Homo sapiens*	NP_003173.1	
Hs-Tac3α (NKB)	*Homo sapiens*	AAQ89042.1	

1. Stewart M, Favrel P, Rotgans B, Wang T, Zhao M, et al., 2014. Neuropeptides encoded by the genomes of the Akoya pearl oyster Pinctata fucata and Pacific oyster Crassostrea gigas: a bioinformatic and peptidomic survey. *BMC Genomics*, 15(1), 840.

2. Siviter RJ, Coast GM, Winther AM, Nachman RJ, Taylor CA, et al., 2000. Expression and functional characterization of a Drosophila neuropeptide precursor with homology to mammalian preprotachykinin A. *J Biol Chem*, 275(30), 23273-80.

3. Siju KP, Reifenrath A, Scheiblich H, Neupert S, Predel R, et al., 2014. Neuropeptides in the antennal lobe of the yellow fever mosquito, Aedes aegypti. J Comp Neurol, 522(3), 592-608.

4. Roller L, Yamanaka N, Watanabe K, Daubnerova I, Zitnan D, et al., 2008. The unique evolution of neuropeptide genes in the silkworm Bombyx mori. Insect Biochem Mol Biol, 38, 1147-1157.

5. Schoofs L, Holman GM, Hayes TK, Nachman RJ, De Loof A, 1990. Locustatachykinin I and II, two novel insect neuropeptides with homology to peptides of the vertebrate tachykinin family. FEBS Lett, 261, 397-401.

6. Hou L, Jiang F, Yang PC, Wang XH, Kang L, 2015. Molecular characterization and expression profiles of neuropeptide precursors in the migratory locust. Insect Biochem Mol Biol, 63, 63-71.

7. Schoofs L, Holman GM, Hayes TK, Kochansky JP, Nachman RJ, et al., 1990. Locustatachykinin III and IV: two additional insect neuropeptides with homology to peptides of the vertebrate tachykinin family. Regul Pept, 31, 199-212.

8. Kanda A, Iwakoshi-Ukena E, Takuwa-Kuroda K, Minakata H, 2003. Isolation and characterization of novel tachykinins from the posterior salivary gland of the common octopus Octopus vulgaris. Peptides, 24(1), 35-43.

9. Ruder T, Ali SA, Ormerod K, Brust A, Roymanchadi ML, et al., 2013. Functional characterization on invertebrate and vertebrate tissues of tachykinin peptides from octopus venoms. Peptides, 47, 71-76.

10. Champagne DE, Ribeiro JM, 1994. Sialokinin I and II: vasodilatory tachykinins from the yellow fever mosquito Aedes aegypti. Proc Natl Acad Sci U S A, 91(1), 138-42.

11. Erspamer V, Anastasi A, 1962. Structure and pharmacological actions of eledoisin, the active endecapeptide of the posterior salivary glands of eledone. *Experientia*, 18(2), 58-59.

Supplementary Table 2. NCBI accession numbers of the receptor sequences used for the phylogenetic analysis shown in Figure 3.

**Sequence Name**	**Species**	**Accession number**
Hdh-TKRL	*Haliotis discus hannai*	MW810094
Hdh-TKRS	*Haliotis discus hannai*	MW929758
*O.vulgaris*_TKR	*Octopus vulgaris*	AB096700
*A.californica*_TKR	*Aplicia californica*	XP_012936180.2
*C.gigas*_TKR	*Crassostrea gigas*	MF320350.1
*M.galloprovincialis*_TKR3	*Mytilus galloprovincialis*	VDI02049.1
*C.teleta*_TKR	*Captitella teleta*	ELT98449.1
*U.unicinctus*_TKR	*Urechis unicinctus*	AB050456.1
*T.urticae*_TKR	*Tetranychus urticae*	XP_01781284.1
*B.dorsalis*_TKR	*Bactrocera dorsalis*	XP011198776
*D.melanogaster*_TKR	*Drosophila melanogaster*	X62711.1
*T.castaneum*_TKR	*Tribolium castaneum*	XP_008194527.2
*A.mellifera*_TKR	*Apis mellifera*	NP_001314884.1
*P.americana*_TKR	*Periplaneta americana*	ARK07245.1
*A.gambiae*_TKR	*Anopheies gambiae*	CAD27763.1
*B.mori*_TKR	*Bombyx mori*	NP_001127722.1
*M.separata*_TKR	*Mythimna separata*	QBA82178.1
*V.destructor*_TKR	*Varroa destructor*	XP_022673516.1
*A.rubens*_TKR1	*Asterias rubens*	XP_033644232.1
*A.rubens*_TKR2	*Asterias rubens*	AVG23011.1
*C.elegans*_TKR1	*Caenorhabditis elegans*	NP_499064.2
*C.intestinalis*_TKR	*Ciona intestinalis*	AB175739.1
*B.floridae*_TKR	*Branchiostoma floridae*	XP_035671126.1
*H.sapiens*_NK2R	*Homo sapiens*	AAB20303.1
*M.musculus*_NK2R	*Mus musculus*	NP_033340.3
*D.rerio*_Tacr2	*Danio rerio*	BX530068.35
*G.gallus*_NK1R	*Gallus gallus*	NP_990199.1
*H.sapiens*_NK1R	*Homo sapiens*	AAA59936.1
*M.musculus*_NK1R	*Mus musculus*	NM_009313.5
*D.rerio*_Tacr1a	*Danio rerio*	JQ924414.1
*D.rerio*_Tacr1b	*Danio rerio*	JQ924415.1
*D.rerio*_Tacr3a	*Danio rerio*	JF317292.1

*D.rerio*_Tacr3b	*Danio rerio*	JF317293.1
*D.rerio*_Tacr3c	*Danio rerio*	XP_002666594
*G.gallus*_NK3R	*Gallus gallus*	NP_001305383.1
*H.sapiens*_NK3R	*Homo sapiens*	M89473.1
*M.musculus*_NK3R	*Mus musculus*	BC066845.1
*V.destructor*_NTLR	*Varroa destructor*	AKR03971.1
*T.urticae*_NTLR	*Tetranychus urticae*	XM_015934988.2
*B.mori*_NTLR (A33)	*Bombyx mori*	NP_001127749.1
*A.gambiae*_NTLR	*Anopheies gambiae*	XP_312088.4
*B.dorsalis*_NTLR	*Bactrocera dorsalis*	XM_029550348.1
*D.melanogaster*_NTLR	*Drosophila melanogaster*	AAA28722.1
*T.castaneum*_NTLR	*Tribolium castaneum*	EEZ99366.1
*B.mori*_NTLR (A32)	*Bombyx mori*	NP_001127748.1
Hdh-sNPFR	*Haliotis discus hannai*	OL907301
*C.tel*_sNPFR	*Capitella teleta*	ELT88594.1
*D.mel*_sNPFR-A	*Drosophila melanogaster*	NP_524176.1
*B.mor*_sNPFR_GPR-A11	*Bombyx mori*	NP_001127708.1

Supplementary Table 3. Half-maximal concentrations (EC50) of Hdh-TK peptides for Hdh-TKRS activation

Peptide	EC50 (nM)	Reporter
Hdh-TK1-1	-	CRE-Luc
Hdh-TK1-2	187.0	
Hdh-TK1-3	15.1	
Hdh-TK2-1	180.5	
Hdh-TK2-2	540.6	
Hdh-TK1-1	-	SRE-Luc
Hdh-TK1-2	-	
Hdh-TK1-3	171.0	
Hdh-TK2-1	3345.0	
Hdh-TK2-2	33.0	

Supplementary Table 4. Half-maximal concentrations (EC50) of Hdh-TK peptides for Hdh-TKRL activation

Peptide	EC50 (nM)	Reporter
Hdh-TK1-1	-	CRE-Luc
Hdh-TK1-2	1039.0	
Hdh-TK1-3	284.0	
Hdh-TK2-1	1102.0	
Hdh-TK2-2	55.0	
Hdh-TK1-1	3672.0	SRE-Luc
Hdh-TK1-2	1147.0	
Hdh-TK1-3	155.0	
Hdh-TK2-1	141.2	
Hdh-TK2-2	285.6	
